# Human coelomic fluid investigation: A MS-based analytical approach to prenatal screening

**DOI:** 10.1038/s41598-018-29384-9

**Published:** 2018-07-20

**Authors:** Donatella Aiello, Antonino Giambona, Filippo Leto, Cristina Passarello, Gianfranca Damiani, Aurelio Maggio, Carlo Siciliano, Anna Napoli

**Affiliations:** 1 Department of Chemistry and Chemical Technologies, University of Calabria, Via P. Bucci, Cubo12/D, I-87036 Arcavacata di Rende (CS), Italy; 2 Department of Pharmacy, Health and Nutritional Sciences, University of Calabria, I-87036 Arcavacata di Rende (CS), Italy; 3 Unit of Hematology for Rare Diseases of Blood and Blood-forming Organs, Regional Reference, Laboratory of Rare Diseases Molecular Diagnosis, Palermo, Italy; 4 U.O.S. D Terapia Fetale e Diagnosi Prenatale, Palermo, Italy

## Abstract

Coelomic fluid (CF) is the earliest dynamic and complex fluid of the gestational sac. CF contains maternal cells and proteins produced by embryonic cells, tissues and excretions. The biochemical composition of CF is modified throughout the first trimester of pregnancy and its protein profile reflects both physiological/pathological changes affecting the embryo and mother. Identification of variations in the balance of proteins might indicate particular types of pathologies, or ascertain specific genetic disorders. A platform utilizing protein enrichment procedures coupled with shotgun identification and iTRAQ differentiation provided the identification and quantitation of 88 unique embryonic proteins. It is relevant to note that chromosome X protein CXorf23 was found suggesting the embryo sex. Foetal sex was determined by Quantitative Fluorescent Polymerase Chain Reaction (QF-PCR) on coelomic cells, foetal tissues and maternal white blood cells, with a 100% concordance rate between iTRAQ-MS/MS and QF-PCR data. The functional associations among the identified proteins were investigated using STRING database. Open Targets Platform showed as significant the following therapeutic areas: nervous, respiratory, eye and head system disease.

## Introduction

Coelomic fluid (CF) is the earliest fluid of the gestational sac, contained into the exocoelomic cavity (ECC) and it is in direct contact with placental villi during the first trimester of pregnancy^1^. Similarly to amniotic fluid (AF), CF might be amenable to prenatal testing. CF is currently used uniquely in genetic screening^2^. At the end of the fourth week of gestation, the ECC splits the extraembryonic mesoderm into two layers, the splanchnic mesoderm lining the secondary yolk sac and the embryo, and the somatic mesoderm lining the trophoblast^3^. The ECC is the largest space inside human gestational sac until the 9th week of gestation, after that time it starts contracting, while the amniotic cavity expands^4^. The anatomical location of ECC suggests its important role played in foetus nutrition, before the placental circulation is established^5^. CF contains adequate amounts of foetus cells, together proteins from embryonic tissues and excretions, with a low rate of contamination of the sample by maternal cells^6^. Similarly to other biological fluids, CF contains several proteins produced by the villous trophoblast, molecules that are transferred through the trophoblastic barrier (i.e. amino acids^7^, antioxidants, acids, metabolites^8^, etc.), some foetal proteins, and proteins synthesized by the secondary yolk sac, which is connected with foetal digestive and vascular systems^3,9–11^. In the first trimester of gestation, the CF protein concentration increases^12^. This alteration is not directly influenced by changes of maternal serum protein concentration, being rather closely dependent on the availability of a constant supply of amino acids for protein synthesis. Protein profile might reflect foetus and maternal physiological/pathological changes and its composition is obviously modified during the first trimester of pregnancy^13^. CF can selectively be aspirated under ultrasound guidance using a transvaginal route at 5–8 week gestation^13^. This procedure, called coelocentesis, was pioneered in 1993 by Jurkovic *et al*.^13^ and might be preferable to chorionic villous sampling (CVS), since the time to diagnosis is shortened, the risk of placental vascular damage and related foetal abnormalities are avoided. Moreover, protein profile should limit discordances resulting from pseudo-mosaicism encountered with classical chorionic preparations, facilitating in utero stem-cell therapy before the foetus becomes immunologically competent^14^. From a clinical point of view, detection of specific biomarkers in CF represents an interesting alternative to tissue sampling, due to the less invasive nature of collection^15^. Recently, the application of various advanced MS-based platforms enabled the discovery of novel biomarkers in biological fluids^16^ and tissues^17–19^. Due to their high selectivity and high sensitivity, these MS-based platforms have been widely used for the identification and quantitation of metabolites^20,21^, amino acids and their synthetic and non-natural analogues^22–24^, medicinal materials, proteins in biological fluids and tissues. Mass spectrometry-based shotgun proteomics methodology has become a standard method for characterization of proteomes from biological samples^25,26^, and for the comprehensive understanding of many metabolic processes in living organisms^13,27,28^. Many putative markers for anomalies, as premature rupture of amnion, intra-amniotic infection, and Down syndrome, have been re-analyzed or newly discovered by MS approaches^13,29–32^. Despite the accessibility of gestational fluids and tissues, such as CF, placenta and foetal membranes, MS investigations have seldom been applied in pregnancy research^10,33^. The relatively low number of embryo cells in CF, the necessity to collect only a small amount of this fluid, the risk of contamination of the sample by maternal material might limit molecular biology assays for genetic analyses^34,35^. The aim was to establish a differential proteomic expression profile of CF via a shotgun proteomic workflow. The presence of abundant serum proteins^33,36^ represents a barrier to detection of medium and low abundance proteins in proteomic analyses. This idea prompted us to develop three different analytical strategies for embryonic protein enrichment from normal CF (patients with no karyotype abnormalities) in order to increase the depth of CF proteome identification and to improve sensitivity for targeted analyses of differentially expressed proteins.

A platform utilizing protein enrichment procedures, such as chemical fractionation, hydroxyapatite (HTP), and immune depletion by “ProteoPrepBlu Albumin and IgG depletion Medium” (PROT–BA) and “Multiple Affinity Removal Spin” cartridges (MARS), coupled with shotgun identification and iTRAQ differentiation of CF was exploited. The iTRAQ-MARS was established as the most efficient approach among those tested, and provided the identification and quantitation of 88 unique embryonic proteins. Chromosome X protein CXorf23 was found suggesting embryo sex. To validate the results obtained by proteomic analysis, QF-PCR amplification using several markers for chromosomes X, Y was performed on 22 serial samples of coelomic cells (CC), foetal tissue (FT) and maternal white blood cells (MBC). Multiplex analyses of CC, FT and MBC samples allowed the distinction of foetal from maternal patterns and the identification of maternal contamination of the CF samples. Prenatal detection of feotal sex was successful in all cases. Bioinformatics elaboration of MS data was performed by STRING database and Open Targets Platform (OTP). OTP showed as significant the following therapeutic areas: “nervous system disease”, “respiratory system disease”, “eye disease” and “head disease”.

## Results

Coelomic fluid (CF) is a yellow viscous fluid with lower protein concentrations. Jauniaux *et al*. found that total protein content is 18 times lower in CF than in maternal serum, and 54 times higher in CF than in AF (Supporting information, Table 1S)^3^. Giambona and co-workers adopted a direct micromanipulator pickup of the embryo-foetal cells selected on the basis of their morphology^34,35^ to remove maternal cell contamination and to obtain an early prenatal diagnosis of gene disorders. CF proteome reveals also maternal contamination, and the protein quantification has been reported in the updated literature data with no distinction between embryo and mother^3^. CF shares many proteins with maternal plasma, trophoblastic, and yolk sac^33^. Cindrova-Davies *et al*.^33^ identified 165 proteins from CF using a gel electrophoresis liquid chromatography (GELC)-MS/MS approach. Serum and common circulating blood proteins respectively accounted for 30% and 10% of the total number of identified and categorized proteins. Therefore, depletion of these proteins is a prerequisite for the detection of the low-abundance components. The starting point to overcome the maternal contamination should also require the removal of IgG and IgA, belonging to the mother immune system since the foetus is immunologically not competent, and HSA, playing a nutritional role during the foetal growth. It is now well accepted that no one single proteomic workflow would come even close to identifying all major proteins in action^37^. The introduction of LC-MS/MS or LC-MALDI MS/MS based shotgun proteomics approaches in conjunction with several pre-fractionation schemes has proven to be a valid and complementary alternative to 2-DE gel-based analysis^38,39^.

**Figure 1:**
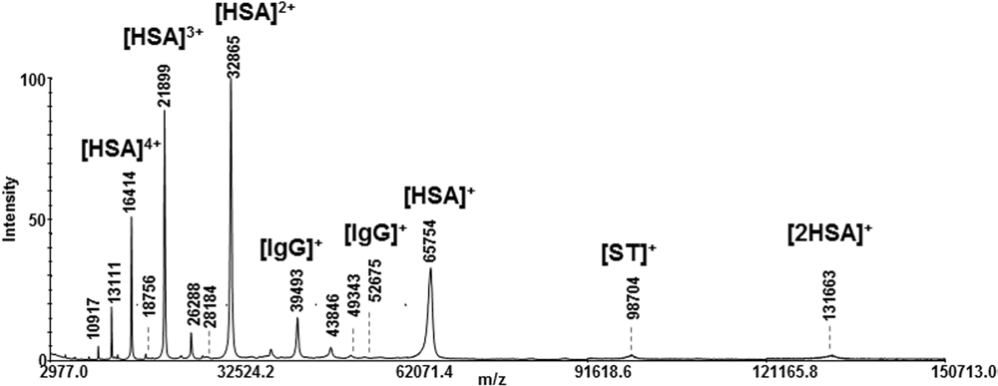
Linear MALDI MS of CF sample.

**Scheme 1:**
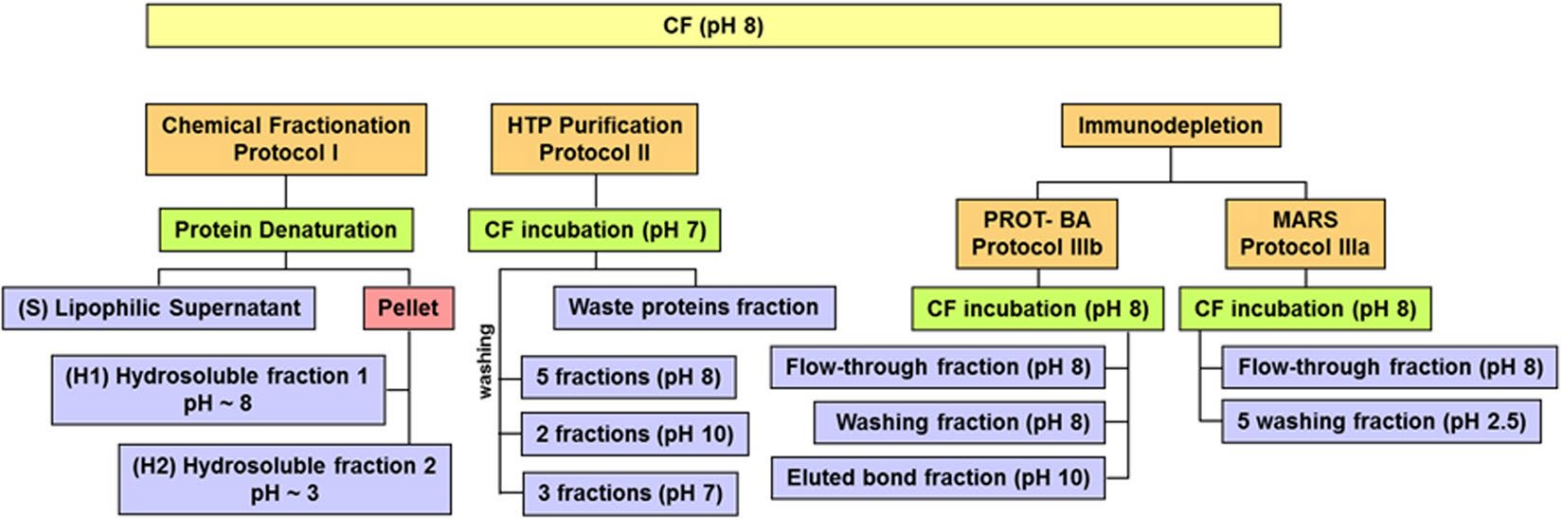
Sample preparation protocols used in this study. Protocol **I**: chemical fractionation; Protocol **II**: HTP purification; Protocols **III**: immunodepletion. All fractions appearing in blue were monitored by SDS-PAGE and/or linear MALDI MS.

The aim was to establish a differential proteomic expression profile of CF *via* a shotgun proteomic workflow. The practicality in the rapid detection of low abundant classes of protein families was used to critically evaluate the pitfalls and strengths of the approach. Three different analytical strategies for embryonic protein enrichment from CF were designed wherein information with respect to the readiness of the protein entry being detected by any one approach were used as an indicator of the specificity. The first step was to establish an efficient methodology to perform, monitor, and compare different pre-fractionation schemes, for the development of a distinctive approach in which efforts were not directed primarily towards identifying markers, but rather in establishing a proteomic expression profile of CF. The protein profile of CF obtained by direct MALDI mass spectrometry in the linear mode showed the presence of multicharged species of HSA (Fig. 1). The five ion peaks of m/z 131663, 65754, 32865, 21899 and 16414 can be ascribed to [2HSA]^+^, [HSA]^+^, [HSA]^2+^, [HSA]^3+^and [HSA]^4+^, respectively. IgG and serotransferrin gave peaks at m/z 52675, 98704, and 39493 respectively. Spectrum showed also the presence of other less intense peaks, nevertheless the attribution was not so simple at this level. Three different protocols were used for sample preparation and variations were “tried-and-tested” within each procedure (Scheme 1, Experimental Section). Protocol I involved a chemical fractionation of proteins carried out by a simple procedure based on their different physicochemical properties. The acid isoelectric point of HSA (IP 5.2) suggested the protein to be soluble under basic conditions. The experimental design was planned in order to obtain three fractions: the supernatant fraction (**S**) and two hydrosoluble fractions (basic **H**_**1**_ and acidic **H**_**2**_). All fractions (**S**, **H**_**1**_ and **H**_**2**_) were directly analyzed by MALDI mass spectrometry and checked by SDS-PAGE (Supporting Information, Figs 1S and 2S).

Analyses showed the presence of albumin and similar protein profile in all fractions, indicating the non-specificity of the method. Finally, the three fractions were subjected to in-solution protein digestion and chromatographic fractionation, then analyzed by MALDI MS/MS. The Protein Pilot software allowed the identification of 104 proteins from fractions **H**_**1**_ and **H**_**2**_ (Supporting Information, Table 2S). Protocol II was based on depletion by home-made HTP spin column^40–43^. HTP is a crystalline form of calcium phosphate which is widely used in biochemistry because its specificity for fractionation and purification of monoclonal antibodies and proteins. HTP chromatography was performed by applying a salt and pH gradient (Fig. 6S, Protocol **II**) in order to obtain an efficient separation according to the different protein isoelectric points^44^. All steps were monitored by SDS-Page and mass spectrometry. MALDI and SDS-Page protein profile of HTP fractions revealed the ubiquitous presence of HSA (Supporting Information, Figs 3S and 4S), demonstrating the non-specificity of the method. In order to check all fractions by direct mass spectrometry, a novel MS-compatible experimental procedure based on the use of immunoaffinity devices was designed (Experimental session)^18^. MARS cartridge (**III**_**a**_**)** can selectively remove high-abundant proteins from human serum, plasma, and cerebrospinal fluid, offering the opportunity to analyze up to 200 samples with no memory effect. The selective immunodepletion provides an enriched pool of low-abundant proteins for downstream proteomics analysis. PROT-BA device (**III**_**b**_) is specific for albumin and IgG depletion from human serum (25–50 μL).The immunoaffinity medium in the prepacked spin column is a mixture of two beaded mediums containing recombinant expressed small single-chain antibody ligands, resulting in low non-specific binding and high capacity.

**Figure 2:**
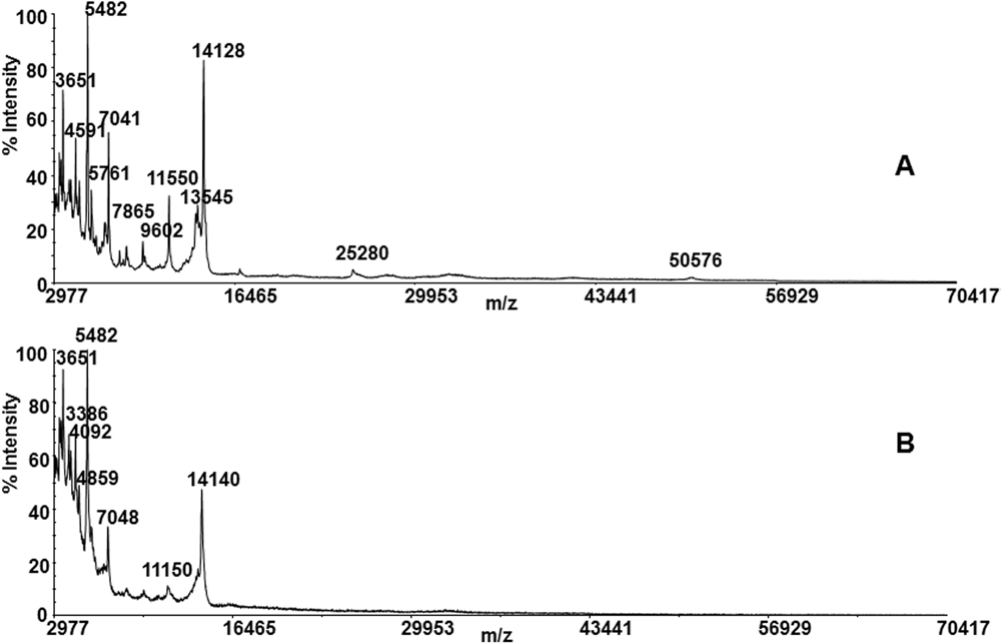
Linear MALDI spectra of CF from Protocol **III**_**a**_ MARS**:** (**A**) after the depletion procedure, and (**B**) after the deglycosylation step by PNGase F.

The depletion efficiency of protocols **III**_**a**_ and **III**_**b**_ was compared by linear MALDI mass spectrometry. MS/MS analysis was used to further evaluate the most efficient setup to deliver the highest number of identified low-abundant proteins. Linear MALDI spectra of fractions collected from PROT-BA showed residual HSA (Supporting information, Fig. 5SA,B). MS/MS analysis of tryptic peptides from the PROT-BA depleted fraction allowed the identification of 48 proteins and several different isoforms (Supporting Information, Table 3S). MARS led to an excellent HSA depletion. Figure 2A shows the linear MALDI spectra of the depleted fraction. In this case, 95 proteins were identified by MS/MS analysis (Table 4S, Supporting Information). Therefore, MARS was reputed to be the best analytical device for CF protein quantitation.

Shotgun identification and iTRAQ differentiation of CF were carried out on twelve individual samples (**DCF**_**A-N**_). To improve the sequence coverage and the number of identified peptides, a deglycosylation step was performed after the MARS protocol application. The iTRAQ-MS/MS analysis of **DCF**_**A-N**_ revealed 88 differentially expressed proteins using an ion ratio ≥2 or ≤0.5. Only 49 UniProtKB validated sequences were evaluated (Table 1). At least three peptides were used to identify and quantify CF proteins. Table 1 lists iTRAQ-MARS differentiation data for individual samples A-C. All experiments were performed in triplicate.

**Table 1:** Proteins quantified by iTRAQ-MARS. Identification and quantification of proteins were performed using the Protein Pilot Paragon Method.

	Accession^a^	Name^a^	Gene Name^a^	B_115_:P_114_^b^	A_116_:P_114_^b^	C_117_:P_114_^b^
1	RM10_HUMAN	39 S ribosomal protein L10, mitochondrial	MRPL10	1.08	1.03	0.66
2	APCL_HUMAN	Adenomatous polyposis coli protein 2	APC2	3.67	6.62	4.88
3	SIA7E_HUMAN	Alpha-N-acetylgalactosaminide alpha-2,6-sialyltransferase 5	ST6GALNAC5	1.42	2.14	1.74
4	RNPL1_HUMAN	Arginyl aminopeptidase-like 1	RNPEPL1	0.21	0.68	0.75
5	ANPRB_HUMAN	Atrial natriuretic peptide receptor 2	NPR2	1.38	3.67	2.76
6	CTF8A_HUMAN	Chromosome transmission fidelity protein 8 homolog isoform 2	CHTF8	2.44	4.11	3.30
7	CC183_HUMAN	Coiled-coil domain-containing protein 183 (Isoform 4)	CCDC183	2.35	2.05	1.43
8	CC186_HUMAN	Coiled-coil domain-containing protein 186	CCDC186	1.55	3.86	1.94
9	CO5A1_HUMAN	Collagen alpha-1(V) chain	COL5A1	1.98	3.36	2.92
10	CR2_HUMAN	Complement receptor type 2 (Isoform D)	CR2	1.81	3.22	2.47
11	NEUL1_HUMAN	E3 ubiquitin-proteinligase NEURL1	NEURL1	1.49	3.96	2.57
12	FLNC_HUMAN	Filamin-C	FLNC	1.23	3.94	3.93
13	GRASP_HUMAN	General receptor for phosphoinositides 1-associated scaffold protein (isoforms 2)	GRASP	1.24	2.56	2.20
14	GLHA_HUMAN	Glycoprotein hormones alpha chain	CGA	1.52	1.71	1.72
15	GCC2_HUMAN	GRIP and coiled-coil domain-containing protein 2	GCC2	1.61	4.83	3.08
16	H3C_HUMAN	Histone H3.3C	H3F3C	2.06	4.63	3.39
17	HV311_HUMAN	Ig heavy chain V-III region KOL	IGHV3–33	1.37	2.52	0.81
18	IPO8_HUMAN	Importin-8	IPO8	1.36	1.60	1.73
19	G137C_HUMAN	Integral membrane protein GPR137C	GPR137C	2.00	4.20	2.60
20	ITIH1_HUMAN	Inter-alpha-trypsin inhibitor heavy chain H1	ITIH1	2.02	1.34	1.31
21	DCP2_HUMAN	m7GpppN-mRNA hydrolase	DCP2	1.10	3.52	2.50
22	MPEG1_HUMAN	Macrophage-expressed gene 1 protein	MPEG1	2.33	6.14	2.93
23	KISS1_HUMAN	Metastasis-suppressor KiSS-1	KISS1	3.38	2.63	1.64
24	MIIP_HUMAN	Migration and invasion-inhibitory protein	MIIP	2.36	4.22	3.47
25	MINT_HUMAN	Msx2-interacting protein	SPEN	1.73	3.85	4.55
26	MUC16_HUMAN	Mucin-16	MUC16	2.40	5.37	4.02
27	MUC19_HUMAN	Mucin-19	MUC19	1.87	4.26	2.44
28	MCTP1_HUMAN	Multiple C2 and transmembrane domain-containing protein 1	MCTP1	3.11	7.33	2.57
29	ACM1_HUMAN	Muscarinic acetylcholine receptor M1	CHRM1	1.00	2.49	1.43
30	NDUA5_HUMAN	NADH dehydrogenase [ubiquinone] 1 alpha subcomplex subunit 5 (Isoform2)	NDUFA5	1.09	2.85	3.08
31	PK3CG_HUMAN	Phosphatidylinositol 4,5-bisphosphate 3-kinase catalytic subunit gamma isoform	PIK3CG	2.36	2.39	1.86
32	PMF1_HUMAN	Polyamine-modulated factor 1 (Isoform 2)	PMF1	1.40	0.00	0.86
33	PSME2_HUMAN	Proteasome activator complex subunit 2	PSME2	1.50	5.20	1.24
34	K1456_HUMAN	Putative methyltransferase KIAA1456	KIAA1456	1.57	2.94	1.97
35	SHSA8_HUMAN	Putative protein shisa-8	SHISA8	2.24	4.32	2.90
36	RANB9_HUMAN	Ran-binding protein 9 (Isoform 2)	RANBP9	2.28	2.35	2.82
37	RALB_HUMAN	Ras-related protein Ral-B	RALB	0.26	1.28	0.07
38	EWS_HUMAN	RNA-binding protein EWS	EWSR1	1.03	1.89	1.06
39	SPCS3_HUMAN	Signal peptidase complex subunit 3	SPCS3	1.33	3.49	2.63
40	SLF2_HUMAN	SMC5-SMC6 complex localization factor protein 2	SLF2	1.29	0.78	2.63
41	SPEG_HUMAN	Striated muscle preferentially expressed protein kinase	SPEG	1.30	3.85	1.41
42	TPC12_HUMAN	Traffickingproteinparticlecomplexsubunit 12	TRAPPC12	0.81	2.30	0.99
43	TRIM3_HUMAN	Tripartite motif-containing protein 3 (Isoform Gamma)	TRIM3	3.14	6.92	4.49
44	UBP37_HUMAN	Ubiquitin carboxyl-terminal hydrolase 37	USP37	1.54	2.48	1.58
45	CX023_HUMAN	Uncharacterized protein CXorf23	CXorf23	1.90	3.49	2.91
46	WDR97_HUMAN	WD repeat-containing protein KIAA1875	WDR97	2.78	2.42	3.45
47	XKR7_HUMAN	XK-related protein 7	XKR7	0.95	2.47	2.07
48	ZC11A_HUMAN	Zinc finger CCCH domain-containing protein 11A	ZC3H11A	2.17	4.67	2.06
49	ZN592_HUMAN	Zinc finger protein 592	ZNF592	2.96	6.72	6.03

The MS/MS data were processed using a mass tolerance of 10 ppm and 0.2 Da for the precursor and fragment ions, respectively. ^a^According to “UniProtKB” (http://www.uniprot.org/). ^b^The relative quantification was calculated according to Protein Pilot, and based on the ratio of the reporter ions corresponding to the **A**_**116**_, **B**_**115**_ and **C**_**117**_ tryptic peptides, over the ratio of the **P**_**114**_ reporter ions. Proteins giving tryptic peptides with an average reporter ion ratio ≥2 or ≤0.5 were classified as up- or down-regulated, respectively.

### Quantitative Fluorescent Polymerase Chain Reaction (QF-PCR). Evaluation of foetal sex

**Figure 3:**
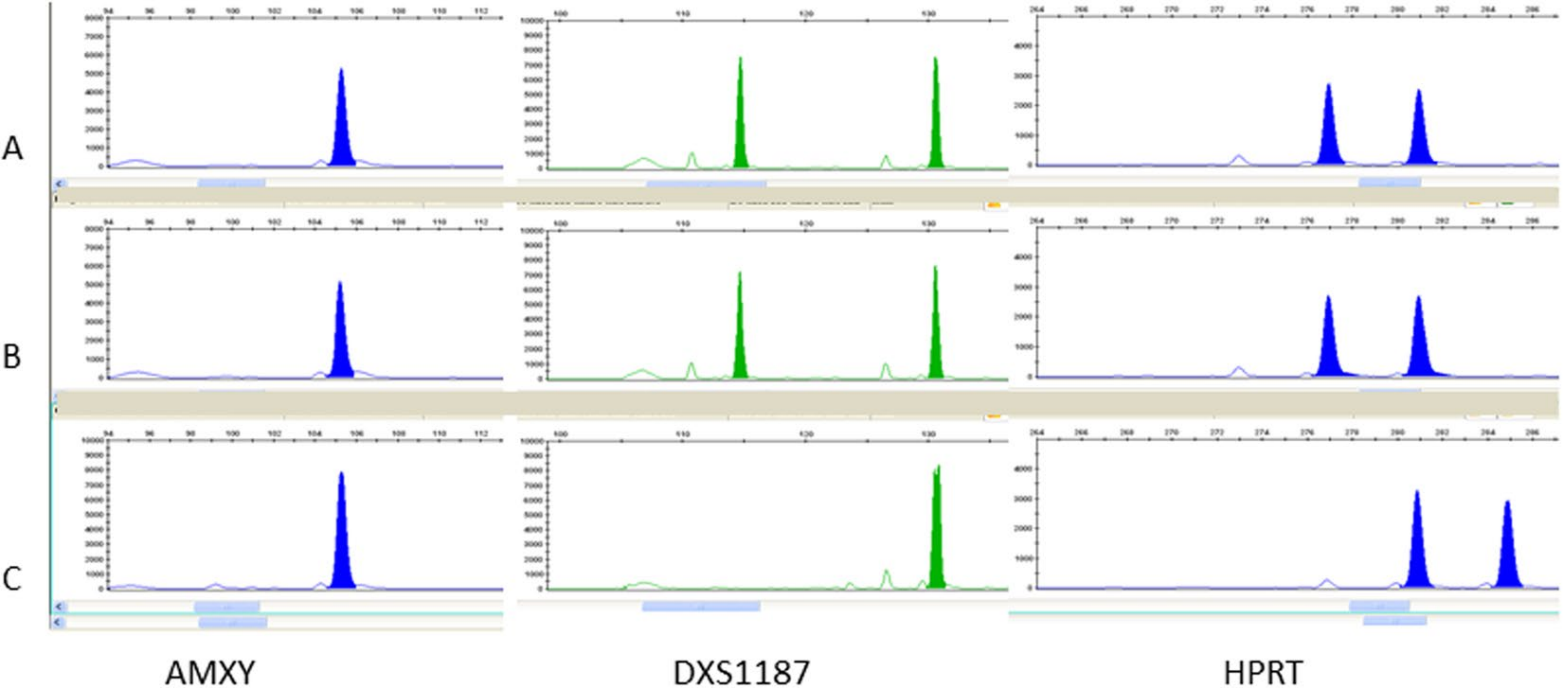
Coelomic fluid of sample A. Evaluation of CF for sex determination by quantitative fluorescent PCR. Electropherograms of amplification product from CF without maternal contamination using small tandem repeat (STR) markers specific for chromosome X. A part of the total electropherograms is displayed in Panel A–C (**A**) coelomic cells pattern; (**B**) foetal tissue pattern; (**C**) maternal pattern. AMXY: is present with a peak of 105 bp; DXS1187: STR located in the X chromosome. Coelomic cell DNA and foetal DNA showed two X peaks, one of maternal origin and one of paternal origin. HPRT: STR located in the X chromosome. Coelomic cell DNA and foetal DNA showed two X peaks, one of maternal origin and one of paternal origin.

**Figure 4:**
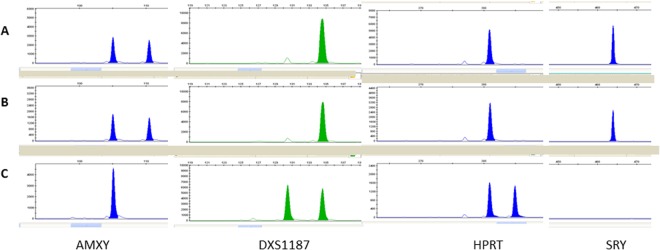
Coelomic fluid of sample B. Evaluation of CF for sex determination by quantitative fluorescent PCR. Electropherograms of amplification product from CF without maternal contamination using small tandem repeat (STR) markers specific for chromosome Y. A part of the total electropherograms are displayed in Panel A–C (**A**) coelomic cell pattern; (**B**) foetal tissue pattern; (**C**) maternal pattern. AMXY: are visible two different peaks, one peak of 105 bp specific for X chromosome, and one of 110 bp specific for Y chromosome. DXS1187: STR located in the X chromosome. Coelomic cell DNA and foetal DNA showed only one peak of maternal origin. HPRT: STR located in the X chromosome. Coelomic cell DNA and foetal DNA showed only one peak of maternal origin. SRY: STR located in the Y chromosome, absent in the profile of the mother and present as one peak in the profiles of coelomic cell DNA and foetal DNA.

Embryo-foetal nucleated red cells in CF were identified by optical phase contrast microscopy. These are roundish cells with a diameter of 12–16 μm, high cytoplasmic nuclear ratio, and the nucleus polarized to one side of the cell near the wall. Coelomic cells isolated from CF were successfully analyzed to obtain information on foetal sex. Quantitative Fluorescent Polymerase Chain Reaction (QF-PCR) was used to evaluate foetal sex. Specific short tandem repeats (STR) of highly variable chromosomal markers (STR) located on chromosome X and Y chromosomes (AMXY, HPRT, SRY, DXS1187, DXS8377, DXS6803, DXS6809) were used to obtain information on foetal sex. Figures 3 and 4 display parts of electropherograms of QF-PCR polymorphic STR markers for samples from a female and a male foetus. Specific markers of X and Y chromosomes are showed. Patterns of DNA derived from coelomic cells, fetal tissues and maternal white blood cells are reported (Figs 3 and 4).

## Discussion

**Figure 5:**
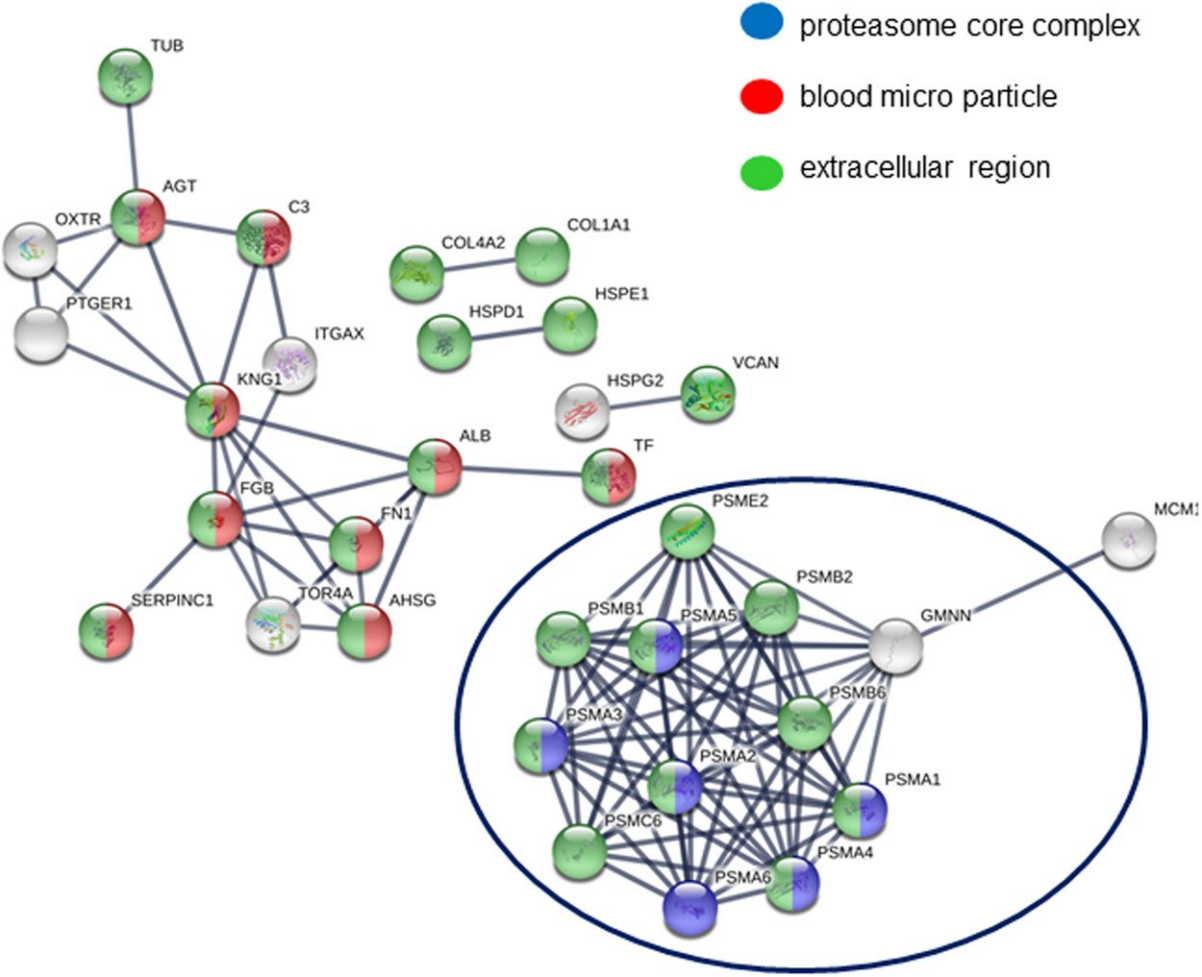
Interaction network analysis of proteins identified by iTRAQ-MARS MS/MS approach (STRING database).

Prenatal screening tests play an important role in the diagnosis for affected pregnancies. Pregnancy progression and birth involve foetal/maternal biochemical processes that depend on complex interactions at multiple levels. The balance among these interactions is disturbed at more than one level when a major problem arises. Proteins represent the functional complements of genes, therefore disorders, as well as changes in the number of gene copies and/or gene regulatory mechanisms, are reflected at the level of protein production and expression. The complex nature of biological fluids requires well established analytical methodologies for the enrichment of low abundance proteins or efficient sample fractionation steps before proteomics analysis. There are no currently preferred or standardized protocols to separate proteins from body fluid proteomes, and gel electrophoresis and chromatography are considered to be complementary. Characterization of CF proteome might be a key point for an innovative prenatal screening. CF is quite different in comparison to the other biological fluids which are usually adopted for clinical screening (plasma, serum, urine). The protein content of CF is profusely due to the maternal contamination, that include serum albumin and immunoglobulins in high concentrations (1.7 g/L and 35 mg/L, respectively)^3^. Therefore, immunodepletion avoids the masking effect of high abundance proteins for obtaining information about the whole CF proteome. Protocols **I** and **II** were planned taking into account the physico-chemical properties of HSA. Protocol **I** led to the identification of 104 proteins, distributed between acidic and basic proteins. The most part of proteins were identified from only one or three peptides, and the method showed a low specificity towards HSA. Moreover, several isoforms and thirty peptides of HSA were also detected, confirming the masking effect exerted by high abundance proteins. In protocol **II**, based on the use of HTP resin, a series of gradients were applied in order to achieve an efficient protein separation. The elution of basic and acidic proteins required the use of KCl, TRIS and EDTA solutions which caused difficult in monitoring all fractions by MALDI mass spectrometry. The fractions collected from the column were pooled according to the elution profile and analyzed by SDS-PAGE. The last fractions eluted by water were checked by mass spectrometry. Spectra revealed the HSA isolation to be unsuccessful, confirming the non-specificity of the method (Figs 3S and 4S, Supporting Information). The other two protocols (**III**_**a**_ and **III**_**b**_), based on the use of immunoaffinity devices, yielded the best results. Notwithstanding, the PROT-BA method was characterized by low recovery of the total protein content, and the nature as mono use device limited its practicality. Under the adopted experimental conditions, the non-complete depletion of HSA was observed and only 48 proteins were identified (Fig. 5S and Table 3S, Supporting Information). HSA removal was strongly improved by the MARS protocol and this method provided the identification of 94 proteins. The reusability of the same device without memory effects was considered striking. The MARS protocol was then adopted for the quantitative analysis. The use of the deglycosylating enzyme PNGase F was a ploy to improve the number of detectable peptides for protein sequence coverage. Figure 2B shows several little mass shifts related to sugar removal from proteins. The CF develops during the 4th week of gestation, and it can be aspirated starting from the 5th week making coelocentesis the earliest possible method of prenatal diagnosis. It is reasonable to consider coelocentesis to be a source of foetal progenitor and stem cells, and CF to be associated to the foetal system^45^. The proteomics outlook becomes a complementary and fundamental source of information. Quantitative proteomics analysis of CF might open an ideal route to the discovery of more efficient and specific biomarkers, elucidating changes mostly related to physiological and pathological conditions in the foetus in the first trimester of pregnancy. The iTRAQ-MARS protocol provided the identification and quantification of 88 proteins, 49 having reviewed UniProtKB sequences (Table 1). It is worthy of note that literature reports realistic associations between specific diseases recognized by maternal serum/plasma or amniotic fluid diagnostics and several proteins detected and quantified by our iTRAQ-MARS protocol. APC2 (Adenomatous polyposis coli protein 2, APCL_HUMAN; Table 1, lane 2) was identified in maternal plasma and amniotic fluid by LC-MS/MS. APC2 is involved in actin-associated events influencing cell motility or adhesion through interaction with actin filaments^46^. Mutations of the gene expressing this protein are related to a form of Sotos syndrome, characterized by prenatal and postnatal childhood overgrowth, developmental delay, mental retardation, advanced bone age, and abnormal craniofacial morphology. Fragments of COL5A1 (Collagen alpha-1(V) chain, CO5A1_HUMAN; Table 1, lane 8), have been found up-regulated in amniotic fluid from patients in pregnancies with Down Syndrome affected foetus^30^. ITIH1 (Inter-alpha-trypsin inhibitor heavy chain H1, ITIH1; Table 1, lane 17) has been characterized by LC-MS and predictive analysis of microarrays, and reported to be correlated to pregnancy-related complications, such as pre-eclampsia and preterm birth^47^. Since the analyzed samples were from patients with no karyotype abnormalities, at the moment it is not possible to establish strict relationships with diseases. However, it is important to note that chromosome X protein CXorf23 (Uncharacterized protein CXorf23, CX023_HUMAN; Table 1, lane 45) was found over-expressed in samples**A**_**116**_ and **C**_**117**_, suggesting an embryo female sex. The same protein was found down regulated in sample **B**_**115**_. Genomic DNA analysis from corionic villus and maternal blood confirmed the sex of foetus; in particular, female for **A**_**116**_ and **C**_**117**_, and male for **B**_**115**_, proving that CF can certainly be used in prenatal screening. The proteomic approach used here led to a differential identification of the catalogue of CF proteins. These data can further be used for bioinformatics elaboration. The biological associations among the identified proteins were investigated using the STRING database. The predicted protein-protein associations were queried through a vast number of databases derived in different ways (e.g., experimentally determined interactions, protein neighborhood data, or data acquired via text mining)^48^.

**Figure 6:**
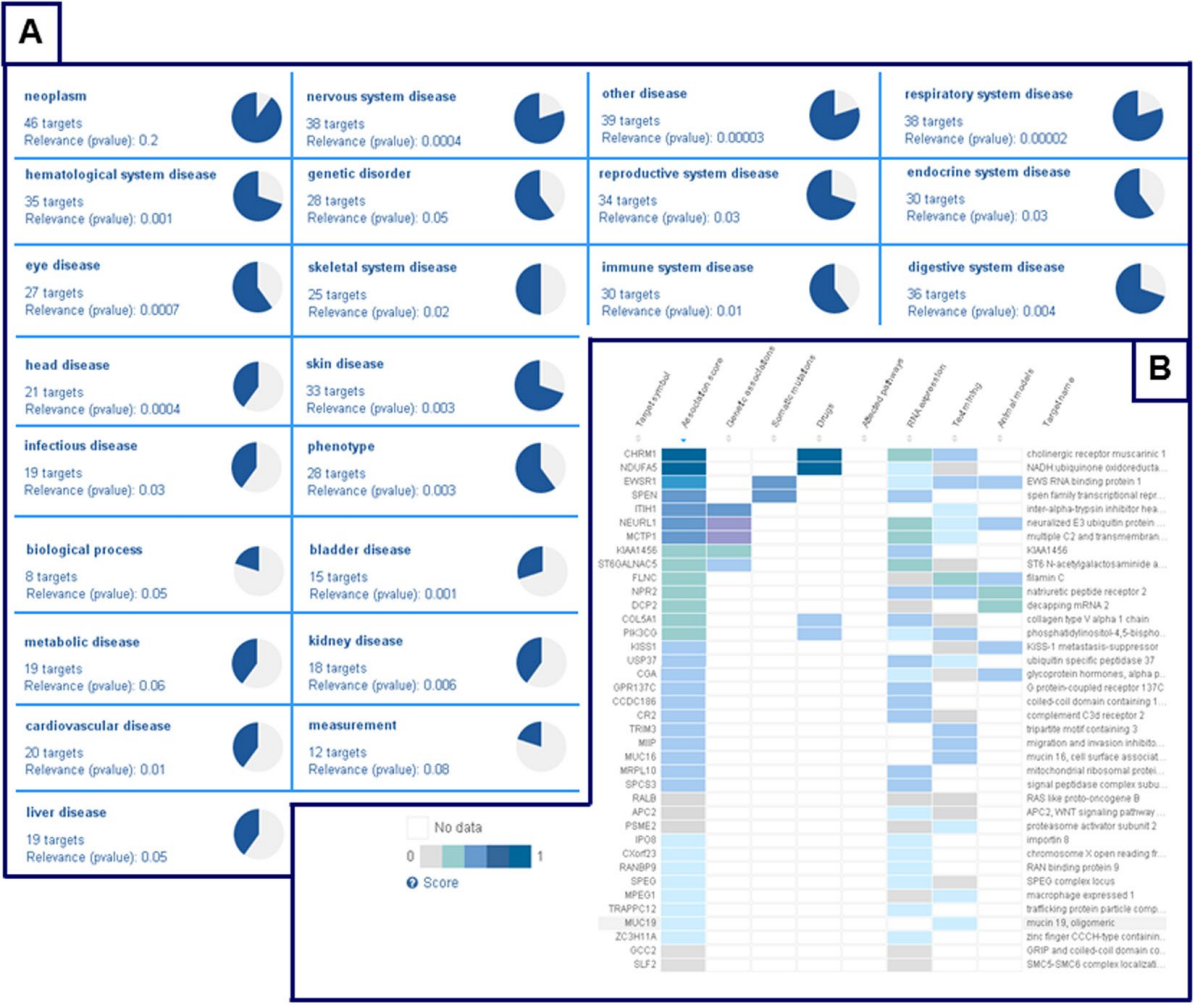
Open Target Platform output. (**A**) Summary page for 49 targets. (**B**) Platform workflow for nervous system diseases.

As shown in Fig. 5 three main networks of cellular components (GO) were identified: proteasome core complex (GO:0019773, blu), blood micro particle (GO:0072562, red), and extracellular region (GO:0005576, green). For the 49 differentially expressed proteins, functional enrichment analysis showed two major pathways in INTERPRO Protein domains and features networks: proteasome, subunit alpha/beta (IPR001353), nucleophile aminohydrolases, N-terminal (IPR029055) (Table 5S, Supporting Information). It is not surprising to observe the relevance of proteasome in GO and INTERPRO networks because proteasome constitutes the central proteolytic machinery of the highly conserved ubiquitin/proteasome system, the major cellular tool for extralysosomal protein degradation. Proteasome can play opposite roles in the regulation of cell proliferation and apoptosis, these roles are apparently defined by the cell environment and proliferative state. During early embryogenesis, proteasomes perform proteolytic functions and also stored as a maternal supply of proteasomes for the developing embryo. Changes in proteasome distribution during fertilization and further stages of development, could be associated to the replacement of maternal proteasome by proteasomes expressed by the embryo itself^49^. Furthermore, we adopted Open Targets (http://www.opentargets.org) as a tool for the verification of the congruency of results. Open Targets is a public-private partnership to establish an informatics platform, the Target Validation Platform. The Open Targets Platform (OTP) is a comprehensive and robust data integration for access to and visualisation of potential drug targets associated with diseases^50^. A drug target can be a protein, protein complex or RNA molecule, and it is displayed by its gene name according to the Human Gene Nomenclature Committee. OTP links multiple data types and assists users in identifying and prioritizing targets, in our case proteins, for further investigation. We checked a list of 49 targets (Table 1) and the OTP output gave back a summary report, in which therapeutic areas of interest were sorted by relevance to our list. Part of OTP output is reported in Fig. 6, where panel A displays the summary page for 49 targets together with the corresponding therapeutic areas. Some areas did not show correlations with the targets, for example “neoplasm” (p-value 0.2), “liver disease” (p-value 0.05) or “metabolic disease” (p-value 0.06). Nevertheless, other areas showed good correlations with the submitted proteins, for example “nervous system disease” (p-value 0.0004), “respiratory system disease” (p-value 0.00002), “eye disease” (p-value 0.0007) and “head disease” (p-value 0.0004). It is not surprising to observe the relevance of targets related to nervous system disease, since humans have considerably more prenatal maturation of their nervous systems.

Panel B in Fig. 6 displays the summary report obtained for 38 targets related to “nervous system disease”. Colour variations are directly connected with scores. Values of 0.5 and 1 indicate that genes are weakly or strongly involved in diseases, respectively. Several proteins involved in neuronal diseases (Fig. 6, Panel B) were detected, although they can indicate a normal state of the central nervous system (CNS) embryonic evolution. In the first month of gestation specific areas of the CNS begin to form following a sequence of developmental processes including proliferation, migration, differentiation, synaptogenesis, apoptosis, and myelination^51^. In particular, cell precursors of brain and spinal cord in humans start to develop early in embryogenesis, approximately two weeks of gestation, through the process called neurulation. Ending the third week of gestation, the neural folds begin to move together and fuse forming the neural tube, leading to a complete neural tube formation approximately from gestation day 26 to 28. Interruption of neural development during this early period can result in severe abnormalities of the brain and spinal cord.

A quantitative shotgun proteomics analysis strategy was successfully used for the identification and differentiation of embryo proteins from CF. Several putative AF/maternal serum markers were identified. The proposed experimental approach furnished a powerful tool for achieving deeper insight into the CF protein composition in early stage of pregnancy, offering a novel perspective in the investigation of molecular constitution and dynamics of this gestational fluid. However, it should be noted that data reported are specific for gestational ages of 8 weeks and for samples from chromosomally normal pregnancies. In all cases, a concordance rate of 100% was found for sex determination between iTRAQ-MARS and Genomic DNA analysis from corionic villus, or amniotic liquid and maternal blood.

## Methods

### Collection of coelomic fluid

This study is part of an on-going investigation examining the feasibility of analysis on DNA extracted from CF for earlier prenatal diagnosis of foetal diseases. The study was conducted in accordance with the Declaration of Helsinki (Hospital Ethical Committee authorization), protocol number 26-01-2005, No 80 approved by the institutional Review Board of “Ospedali Riuniti Villa Sofia-Cervello”, Palermo, Italia. Informed consent forms were obtained from all the study participants and all methods were performed in accordance with relevant guidelines and regulations. The details of such protocols have been previously described^33,34^. Following written consent, women were recruited between 7–10 weeks of gestation. CF from pregnancies with chromosomally normal foetus was obtained by ultrasound-guided transvaginal puncture, as reported^33,34^. Twenty-two samples of CF were selected for proteomic studies. CF cells were used for morphological and genetic analysis^33,34^, while the fluid was used for proteomic experiments.

### Sample preparation of embryo-foetal cells and Quantitative Fluorescent Polymerase Chain Reaction (QF-PCR)

Embryo-foetal cells were one by one aspirated by a micromanipulator using a 45 μm glass micropipette (BioCare Europe). Cells were placed into a drop of 0.9% NaCl in the same Petri disk. Each drop containing a group of embryo-foetal erythroid precursor cells was centrifuged at 10000 rpm for 7 min and supernatants discarded. All samples were subjected to DNA extraction by alkaline method. QF-PCR was used to evaluate the foetal sex. Specific short tandem repeat (STR) of highly variable chromosomal markers located on X and Y chromosomes (AMXY, HPRT, SRY, DXS1187, DXS8377, DXS6803, DXS6809) were used to obtain information on foetal sex. Each primer was labelled with fluorescent dyes (PET, VIC, NED, 6-FAM) to obtain fluorescent PCR amplicons. DNA (2.5 μL; 10–20 ng) was added to the Master Mix (10 μL), and the mixture was used for amplification according to the manufacturer’s instructions. Denaturation was performed for 15 min at 95 °C, then 26 cycles of 30 s at 95 °C, 90 s at 59 °C, 90 s at 72 °C, and a final extension of 30 min at 72 °C were applied. PCR products (2 μL) were mixed with 15 μL of HiDi and 0.5 μL of LIZ 500 (Applied Biosystems), as the internal-lane size standard. Capillary electrophoresis was performed by an automated ABI Prism 3130 Genetic Analyzer and data were analyzed by the Gene Mapper 4.0 software (Applied Biosystems). Genomic DNA was analyzed from corionic villus or amniotic liquid and maternal blood. DNA extraction was carried out by standard protocols.

### Sample preparation of coelomic fluid proteome

Individual samples of CF were used to check sample preparation procedures (Protocols **I–III**, Scheme 1). An aliquot of 200 μL from ten CF samples were pooled to get analytical robustness and consistence (CFp, pooled coelomic fluid). Individual CF (twelve) was manipulated for the comparative quantification experiments.

### Protocol I

CF (200 μL) was precipitated with 600 μL of CHCl_3_/CH_3_OH 1:3 (v/v), yielding the supernatant fraction **S** and a pellet, which was successively partitioned with 200 μL of 50 mM NH_4_HCO_3_ (**H**_**1**_), and 50 μL of H_2_O/CH_3_CN 3:2 (v/v, TFA 0.3%) (**H**_**2**_).

### Protocol II

Hydroxyapatite (100 mg) was packed in a spin column. CF (200 μL) was added together with 25 μL of equilibration buffer (10 mM TRIS, 1 mM EDTA, pH 7), and loaded onto HTP spin column. After 30 min of incubation at room temperature, the column was spun at 6000 rpm for 2 min. Bound proteins were eluted with 200 μL of elution buffer (100 mM KCl, 20 mM TRIS, 2 mM EDTA, pH 8) for five times, giving 30 min incubation each time, followed by centrifugation at 6000 rpm for 2 min. The column was then washed with 200 μL of (NH_4_)_2_CO_3_ (pH 10), and with 200 μL of H_2_O.

### Immunodepletion

CF proteins were depleted of high abundant proteins using (a) “Multiple Affinity Removal Spin cartridge-MARS” (Agilent Technologies, Italy), or (b) “ProteoPrepBlu Albumin and IgG depletion Medium” (PROT-BA; Sigma-Aldrich, Italy).

### Protocol III_a_

The cartridge was treated four times with 400 µl of 50 mM NH_4_HCO_3_, (pH 8). CF (200 µl) was applied on column, centrifuged for 2 min at 3000 rpm, then collected. The cartridge was washed with 400 µl of 50 mM NH_4_HCO_3_ and the obtained flow-through fractions were collected and concentrated (50 µl). High abundant proteins were eluted with buffer B (5 times, pH 2.5). The collected fraction was dried, than solubilized with 100 µl of buffer (0.375 M TRIS, 0.1% SDS, pH 8.8).

### Protocol III_b_

The cartridge PROT-BA was treated with 200 μl of 50 mM NH_4_HCO_3_, (pH 8). CF (200 μl) was applied on column and incubated for 10 min. After centrifugation at 3000 rpm for 1 min, the flow-through fraction was collected. The cartridge was washed with 200 μl of 50 mM NH_4_HCO_3_ and the collected flow-through fractions were combined. The retained proteins were eluted with (NH_4_)_2_CO_3_ (pH 10), after 10 min of incubation and centrifugation at 3000 rpm for 2 min.

### Tryptic digestion

Each lyophilized fraction was solubilized with 100 µl of 50 mM NH_4_HCO_3_. Trypsin (20 pmol) was then added to the protein mixture, and the digestion step was performed in a home microwave (MWD 246 SL, Whirlpool, Italy) at 250 W irradiation power (12 treatments, each one lasting 2 min). Tryptic peptide mixtures were subjected to reversed phase chromatography fractionation^52^.

### Sample preparation for comparative quantification

200 µL from 10 samples of CF were pooled together (CFp) and used for comparative quantification experiments with twelve individual CFs. CFp and the twelve CFs (A-N) were depleted of high abundant proteins using MARS approach (Protocol **IIIa**).The depleted fractions were collected and dried. The depleted CFp sample (DCFp) was treated with 100 µl of 50 mM NH_4_HCO_3_ and incubated with 4 µL of PNGase F (0.5 unit/μL). The microwave assisted deglycosylation step was performed at 250 W irradiation power (20 treatments each lasting 1 min). The protein mixture was subsequently treated with 200 µl of buffer (0.375 M TRIS, 0.1% SDS, pH 8.8) and boiled in a bain-marie for 5 min. After this time, trypsin (120 pmol) was added. The microwave assisted tryptic digestion was carried as above reported. All tryptic peptide mixtures were purified on a SPE Strata C18-E column (Phenomenex Inc,USA) to eliminate salts^53^ and interferences with the iTRAQ reagent procedure. Column was washed with CH_3_OH, and then conditioned with 2 mL of CH_3_CN/TFA 0.1% (50:50, v/v) and 2 mL of TFA 0.1%. Samples were made acidic by adding 300 µl of TFA 0.5% and loaded on column. The washing step was performed using 4 mL of TFA 0.1%, and the flow-through fractions were wasted. Collection of peptides was performed by using (a) 4 mL of CH_3_CN/TFA 0.1% (50:50, v/v), (b) 4 mL of CH_3_CN/TFA 0.1% (80:20, v/v), and (c) 6 mL of CH_3_CN. The flow-through fractions from steps (a)-(c) were combined and concentrated under vacuum. The dried samples were re-suspended in 50 µl of 500 mM triethyl ammonium bicarbonate buffer (TEAB), and tryptic peptides were labelled with the iTRAQ reagents (m/z 114.1, 115.1, 116.1 and 117.1) following the manufacturer’s protocol. The iTRAQ labels were added to the digested samples, in particular m/z 114.1 reporter ions was added to **DCF**_**P**_ (sample **P**_**114**_), m/z 116.1 reporter ions to **DCF**_**A**,**D**,**G**,**L**_ (samples **A**_**116**_, **D**_**116**_, **G**_**116**_, **L**_**116**_), m/z 115.1 reporter ions to **DCF**_**B**,**E**,**H**,**M**_ (samples **B**_**115**_, **E**_**115**_, **H**_**115**_, **M**_**115**_), and m/z 117.1 reporter ions to **DCF**_**C**,**F**,**I**,**N**_ (samples **C**_**117**_, **F**_**117**_, **I**_**117**_, **N**_**117**_). Labeled samples were combined in four groups (i.e., **P**_**114**_, **A**_**116**_, **B**_**115**_ and **C**_**117**_) and dried prior cation exchange and reversed phase chromatography fractionation. A series of 180 chromatographic fractions were collected for each group.

### MALDI MS and MS/MS analysis

Linear MALDI-TOF spectra were acquired using a 5800 MALDI-TOF/TOF analyzer (AB SCIEX, Germany). All spectra were acquired in default calibration mode averaging 2500 laser shots with a mass accuracy of 500 ppm. MS and MS/MS analyses were performed in reflectron positive-ion mode. All chromatographic fractions were solubilized in 10 µl of matrix (α-CHCA 10 mg/mL, CH_3_CN/0.3% TFA in water, 50:50, v/v). MS spectra were acquired with a laser pulse rate of 400 Hz and at least 4000 laser shots, and CID-MS/MS experiments were performed at collision energy of 1 kV, using ambient air as the collision gas (10^−6^ Torr). CID-MS/MS spectra required up to 5000 laser shots and a pulse rate of 1000 Hz.

### Database Searching and Bioinformatics

Protein identification was performed by the Protein Pilot 4.0 software, using the Paragon protein database search algorithm (AB Sciex)^37^. Data analysis parameters for samples were as follows: Sample type: iTRAQ 4plex (Peptide Labelled); Cys Alkylation: None; Digestion: Trypsin; Instrument: 5800; Special Factors: Phosphorylation emphasis, Species: Homo Sapiens; Quantitate tab: checked; ID Focus: Biological modification and Amino acid substitutions; Database: UniProt_taxonomy_Eukaryota [2759]; Search Effort: Thorough ID; Minimum Detected Protein Threshold [Unused ProtScore (Conf)]: 1 (90.0%); Run. False Discovery Rate Analysis Tab: checked. The relative quantification was based on the ratio of the reporter ions corresponding to the 116.1, 115.1 and 117.1 tryptic peptides, over the ratio of the **P**_**114**_ reporter ions (114.1 Da).

## Electronic supplementary material

Electronic supplementary material

Electronic supplementary material
