# Supplementary material for: Human coelomic fluid investigation: A MS-based analytical approach to prenatal screening
Source: Sci Rep. 2018 Jul 20;8:10973. doi: 10.1038/s41598-018-29384-9 (PMC6054674; doi:10.1038/s41598-018-29384-9)
Supplement: Supplementary file 1 — Electronic supplementary material [file 41598_2018_29384_MOESM1_ESM.doc]

**Human coelomic fluid investigation: A MS-based analytical approach to prenatal screening.**

Donatella Aiello,^±^ Antonino Giambona,^‡^ Filippo Leto,^‡^ Cristina Passarello^‡^, Gianfranca Damiani^¥^, Aurelio Maggio,^‡^ Carlo Siciliano,^§^ Anna Napoli^±*^

±Department of Chemistry and Chemical Technologies, University of Calabria, Via P. Bucci, Cubo12/D, I-87036 Arcavacata di Rende (CS),Italy.

^§^Department of Pharmacy, Health and Nutritional Sciences, University of Calabria, I-87036 Arcavacata di Rende (CS), Italy.

^‡^Unit of Hematology for Rare Diseases of Blood and Blood-forming Organs, Regional Reference, Laboratory of Rare Diseases Molecular Diagnosis, Palermo, Italy.

^¥^U.O.S. D Terapia Fetale e Diagnosi Prenatale, Palermo, Italy.

**Corresponding Author**

*Anna Napoli

Department of Chemistry and Chemical Technologies, University of Calabria, Via P. Bucci, Cubo12/D, I-87036 Arcavacata di Rende (CS), Italy.e-mail:amc.napoli@unical.it; Fax: +39 0984 493307.

**SUPPORTING INFORMATION**

**Table 1S:** Concentration of several proteins in coelomic fluid (CF) and maternal serum (MS) during the first trimester of pregnancy. (Jauniaux, E., Gulbis, B., Jurkovic, D., Schaaps J.P., Campbell, S., Meuris, S. Protein and steroid levels in embryonic cavities of early human pregnacy. Hum. Reprod., 1993, 8, 782-787.)

| **Molecules** | **M.S.** | **C.F.** |
| --- | --- | --- |
| **Mother** |  |  |
| **Total protein (g/l)** | 71.3 | **3.5** |
| Albumin (g/l) | 45.5 | **1.7** |
| Pre-albumin (g/l) | 1.14 | 0.04 |
| Immunoglobulin G (mg/dl) | 907 | **32** |
| Immunoglobulin A (mg/dl) | 122 | **1** |
| Complement factors 3 (mg/dl) | 114 | nd |
| Complement factors 4 (mg/dl) | 21 | nd |
| β2-microglobulin (mg/l) | 0.9 | 4.7 |
| Activin A (ng/ml) | 0.68 | 0.98 |
| Inhibin B (pg/ml) | 5.9 | 24.3 |
| Glucose (mmol/l) | 3.4 | 2.7 |
| **Decidua** |  |  |
| Placental protein 14 (µg/l) | 642 | 4416 |
| Interleukin-6 (ng/ml) | 40 | 88 |
| **Embryo/Fetus** |  |  |
| τ-glutamyltransferase (IU/l) | 9 | 2 |
| Ferritin (µg/l) | 49 | 287 |
| Cancer antigen 125 (IU/ml) | 35 | 35 |

**Figure 1S:** Linear MALDI spectra of chemical fractions from **Protocol I.** (**A**) supernatant fraction **S**; (**B**) basic fraction **H_1_**; (**C**) acid fraction **H_2_**.

**Figure 2S:** Electrophoresis profile of coelomic fluid after chemical fractionation procedure, **Protocol I**.

**Lanes:** **M:** Marker. **1:** coelomic fluid, positive control. **2:** supernatant fraction **S. 3:** fraction **H_1_ (NH_4_HCO_3_, 50 mM). 4:** fraction **H_2_ (H_2_O/CH_3_CN ÷ 3:2 v/v, TFA 0,3%).**

**SDS-PAGE (12.5%).** An homemade protein molecular weight marker containing Lactoferrin (87 kDa, L9507), Bovine Serum Albumin (66 kDa, A2153), Albumin from chicken (44 kDa, A5503), Mioglobin from equine skeletal muscle (17 kDa, M0630) and Cytocrome C (12 kDa,C2506) was loaded on the molecular weight marker lane. Proteins were stained with Comassie Brilliant Blu R-250.

**Table 2S: CHEMICAL FRACTIONATION (Protocol I)**

The identification of proteins was performed using the Protein Pilot Paragon Method. The MS/MS data were processed using a mass tolerance of 10 ppm and 0.2 Da for the precursor and fragment ions, respectively. **^a^**According to “UniProtKB” (http://www.uniprot.org/). **^b^**According to “Compute pI/MW” (http://web.expasy.org/compute_pi/).

|  | **Accession Number^a^** | **Protein Name^a^** | **Gene name^a^** | **IP^b^** | **MW (Da)^b^** |
| --- | --- | --- | --- | --- | --- |
| **1** | O94929_HUMAN | **Actin-binding LIM protein 3** | ABLIM3 | 8.86 | 77,802 |
| **2** | O94929-3_HUMAN | **Actin-binding LIM protein 3** | ABLIM3 | 8.97 | 67.073 |
| **3** | O94929-2_HUMAN | **Actin-binding LIM protein 3** | ABLIM3 | 8.83 | 61,570 |
| **4** | Q8IUK7_HUMAN | **ALB protein** | ALB | 5.77 | 45,160 |
| **5** | C9JKR2_HUMA | **Albumin isoform CRA_k** | ALB | 5.97 | 47,287 |
| **6** | G3V5R8_HUMAN | **Alpha-1-antitrypsin** (fragment) | SERPINA1 | 5.23 | 9,892 |
| **7** | A1AT_HUMAN | **Alpha-1-antitrypsin** (isoform2) | SERPINA1 | 5.26 | 40,263 |
| **8** | P38398_HUMAN | **Breast cancer type 1 susceptibility protein** | BRCA1 | 5.29 | 207,721 |
| **9** | P38398-2_HUMAN | **Breast cancer type 1 susceptibility protein** (isoform2) | BRCA1 | 7.57 | 7,177 |
| **10** | P38398-4_HUMAN | **Breast cancer type 1 susceptibility protein** (isoform4) | BRCA1 | 5.32 | 205,838 |
| **11** | CAR14_HUMAN | **Caspase recruitment domain-containing protein 14** | CARD14 | 5.65 | 113,270 |
| **12** | B4E082_HUMAN | **cDNA FLJ57698, highly similar to Transcription factor p65** | N/A | 8.31 | 47,492 |
| **13** | CO1A1_HUMAN | **Collagen alpha-1(I) chain** | COL1A1 | 5.60 | 138,941 |
| **14** | CO3_HUMAN | **Complement C3** | C3 | 6.02 | 187,148 |
| **15** | DNMT1_HUMAN | **DNA (cytosine-5)-methyltransferase 1** (isoform 2) | DNMT1 | 7.99 | 183,165 |
| **16** | SMBP2_HUMAN | **DNA-binding protein SMUBP-2** | IGHMBP2 | 9.13 | 109,149 |
| **17** | Q9H819-_HUMAN | **DnaJ homolog subfamily C member 18** | DNAJC18 | 7.04 | 41,551 |
| **18** | Q5VZY9_HUMAN | **Doublecortin and CaM kinase-like 1** | DCLK1 | 9.70 | 40,457 |
| **19** | Q96JB1_HUMAN | **Dynein heavy chain 8, axonemal** (Isoform1) | DNAH8 | 5.95 | 514,664 |
| **20** | Q96JB1-2_HUMAN | **Dynein heavy chain 8, axonemal** (Isoform2) | DNAH8 | 5.92 | 510,448 |
| **21** | O94972_HUMAN | **E3 ubiquitin-protein ligase TRIM37** (Isoform1) | TRIM37 | 5.04 | 107,906 |
| **22** | O94972-2_HUMAN | **E3 ubiquitin-protein ligase TRIM37** (Isoform2) | TRIM37 | 5.04 | 89,186 |
| **23** | EPHX4_HUMAN | **Epoxide hydrolase 4** | EPHX4 | 8.64 | 42,324 |
| **24** | FXL22_HUMAN | **F-box/LRR-repeat protein 22** | FBXL22 | 9.99 | 27,269 |
| **25** | FINC_HUMAN | **Fibronectin** | FN1 | 5.46 | 262,625 |
| **26** | NMDE3_HUMAN | **Glutamate [NMDA] receptor subunit epsilon-3** | GRIN2C | 8.82 | 134,209 |
| **27** | B0QZK9_HUMAN | **Heterochromatin protein 1, binding protein 3** (fragment) | HP1BP3 | 4.79 | 8,150 |
| **28** | HV305_HUMAN | **Ig heavy chain V-III region** | IGHV3-13 | 6.54 | 12,506 |
| **29** | INADL_HUMAN | **InaD-like protein** | INADL | 4.83 | 196,368 |
| **30** | INADL_HUMAN | **InaD-like protein** (isoform2) | INADL | 4.84 | 173,793 |
| **31** | INADL_HUMAN | **InaD-like protein** (isoform3) | INADL | 4.80 | 170,238 |
| **32** | INADL_HUMAN | **InaD-like protein** (isoform4) | INADL | 4.81 | 167,159 |
| **33** | INADL_HUMAN | **InaD-like protein** (isoform5) | INADL | 4.78 | 125,231 |
| **34** | DCLK1_HUMAN | **Isoform 1 of Serine/threonine-protein kinase DCLK1** | DCLK1 | 8.84 | 82,224 |
| **35** | FINC_HUMAN | **Isoform 14 of Fibronectin** | FN1 | 5.46 | 262,625 |
| **36** | MTA1_HUMAN | **Metastasis-associated protein MTA1** | MTA1 | 9.34 | 80,786 |
| **37** | F8VZC2_HUMAN | **Microspherule protein 1** | MCRS1 | 11.88 | 6,877 |
| **38** | Q49MG5_HUMAN | **Microtubule-associated protein 9** | MAP9 | 7.59 | 74,234 |
| **39** | MUC19_HUMAN | **Mucin-19** | MUC19 | 4.97 | 805,253 |
| **40** | MSD3_HUMAN | **Myb/SANT-like DNA-binding domain-containing protein 3** | MSANTD3 | 8.58 | 32,363 |
| **41** | NGB_HUMAN | **Neuroglobin** | NGB | 5.38 | 16,933 |
| **42** | E5RJ29_HUMAN | **PH and SEC7 domain-containing protein 3** | PSD3 | 5.73 | 108,990 |
| **43** | PSD3_HUMAN | **PH and SEC7 domain-containing protein 3** (isoform1) | PSD3 | 5.68 | 116,034 |
| **44** | PSD3-2_HUMAN | **PH and SEC7 domain-containing protein 3** (isoform2) | PSD3 | 5.68 | 115,934 |
| **45** | PSD3-3_HUMAN | **PH and SEC7 domain-containing protein 3** (isoform3) | PSD3 | 8.89 | 58,077 |
| **46** | PEDF_HUMAN | **Pigment epithelium-derived factor** | SERPINF1 | 5.97 | 46,312 |
| **47** | Q8N4A0_HUMAN | **Polypeptide N-acetylgalactosaminyltransferase 4** | GALNT4 | 7.55 | 66,666 |
| **48** | F8WDV4_HUMAN | **Protein SSUH2 homolog** | SSUH2 | 10.88 | 4,435 |
| **49** | AG1L2_HUMAN | **Putative glycosyltransferase ALG1L2** | ALG1L2 | 5.47 | 24,154 |
| **50** | RRMJ3_HUMAN | **Putative rRNA methyltransferase 3** | FTSJ3 | 8.53 | 96,558 |
| **51** | F8VXH1_HUMAN | **Rac GTPase-activating protein 1** | RACGAP1 | 11.53 | 6,601 |
| **52** | RAB12_HUMAN | **Ras-related protein Rab-12** | RAB12 | 8.68 | 27,248 |
| **53** | Q6PCB5_HUMAN | **Round spermatid basic protein 1-like protein** | RSBN1L | 8.91 | 94,870 |
| **54** | Q6PCB5-2_HUMAN | **Round spermatid basic protein 1-like protein** (isoform2) | RSBN1L | 5.57 | 65,582 |
| **55** | SACS_HUMAN | **Sacsin** | SACS | 6.63 | 521,126 |
| **56** | B7WNR0_HUMAN | **Serum albumin** | ALB | 6.85 | 56,212 |
| **57** | P02768_HUMAN | **Serum albumin** | ALB | 5.92 | 69,367 |
| **58** | H0YA55_HUMAN | **Serum albumin** | ALB | 6.62 | 51,571 |
| **59** | D6RHD5_HUMAN | **Serum albumin** | ALB | 6.45 | 52,059 |
| **60** | H7BZZ8_HUMAN | **Smoothelin** | SMTN | 9.48 | 23,388 |
| **61** | SPTA2_HUMAN | **Spectrin alpha chain, non-erythrocytic 1** | SPTAN1 | 5.22 | 284,539 |
| **62** | SPTA2_HUMAN | **Spectrin alpha chain, non-erythrocytic 1** (isoform2) | SPTAN1 | 5.23 | 285,094 |
| **63** | SPTA2_HUMAN | **Spectrin alpha chain, non-erythrocytic 1** (isoform3) | SPTAN1 | 5.21 | 282,282 |
| **64** | SYNJ1_HUMAN | **Synaptojanin-1** | SYNJ1 | 7.13 | 173,103 |
| **65** | Q8TB96-_HUMAN | **T-cell immunomodulatory protein** | ITFG1 | 5.15 | 68,108 |
| **66** | TRHDE_HUMAN | **Thyrotropin-releasing hormone-degrading ectoenzyme** | TRHDE | 6.50 | 117,000 |
| **67** | Q04206_HUMAN | **Transcription factor p65** | RELA | 5.46 | 60,219 |
| **68** | E9PI38_HUMAN | **Transcription factor p65** (fragment) | RELA | 8.77 | 20,839 |
| **69** | E9PJR1_HUMAN | **Transcription factor p65** (fragment) | RELA | 8.69 | 9,983 |
| **70** | E9PJZ9_HUMAN | **Transcription factor p65** (fragment) | RELA | 9.78 | 3,271 |
| **71** | E9PKH5_HUMAN | **Transcription factor p65** (fragment) | RELA | 8.65 | 46,697 |
| **72** | E9PKV4_HUMAN | **Transcription factor p65** (fragment) | RELA | 8.73 | 26,652 |
| **73** | E9PM47_HUMAN | **Transcription factor p65** (fragment) | RELA | 7.91 | 7,226 |
| **74** | E9PMD5_HUMAN | **Transcription factor p65** (fragment) | RELA | 8.24 | 17,868 |
| **75** | E9PN69_HUMAN | **Transcription factor p65** (fragment) | RELA | 6.28 | 14,427 |
| **76** | E9PNK5_HUMAN | **Transcription factor p65** (fragment) | RELA | 7.80 | 9,378 |
| **77** | E9PNV4_HUMAN | **Transcription factor p65** (fragment) | RELA | 8.19 | 16,807 |
| **78** | E9PQS6_HUMAN | **Transcription factor p65** (fragment) | RELA | 8.08 | 23,379 |
| **79** | E9PRX2_HUMAN | **Transcription factor p65** (fragment) | RELA | 9.12 | 10,723 |
| **80** | Q2TAM5_HUMAN | **Transcription factor p65** (fragment) | RELA | 8.66 | 42,868 |
| **81** | Q96CP1_HUMAN | **Transcription factor p65** (fragment) | RELA | 6.70 | 27,663 |
| **82** | Q04206-2_HUMAN | **Transcription factor p65** (Isoform2) | RELA | 5.62 | 58,807 |
| **83** | Q04206-3_HUMAN | **Transcription factor p65** (Isoform3) | RELA | 5.72 | 59,068 |
| **84** | Q04206-4_HUMAN | **Transcription factor p65** (Isoform4) | RELA | 5.46 | 59,910 |
| **85** | TRPM6_HUMAN | **Transient receptor potential cation channel subfamily M member 6** | TRPM6 | 7.86 | 231,708 |
| **86** | Q9BX84-6_HUMAN | **Transient receptor potential cation channel subfamily M member 6** (isoform6) | TRPM6 | 7.05 | 97,4520 |
| **87** | Q9BX84-5_HUMAN | **Transient receptor potential cation channel subfamily M member 6** (isoform5) | TRPM6 | 7.60 | 111,301 |
| **88** | Q9BX84-4_HUMAN | **Transient receptor potential cation channel subfamily M member 6** (isoform4) | TRPM6 | 8.17 | 223,124 |
| **89** | Q9BX84-3_HUMAN | **Transient receptor potential cation channel subfamily M member 6** (isoform3) | TRPM6 | 7.86 | 230,956 |
| **90** | Q9BX84-2_HUMAN | **Transient receptor potential cation channel subfamily M member 6** (isoform2) | TRPM6 | 7.94 | 231,041 |
| **91** | TMC5A_HUMAN | **Transmembrane and coiled-coil domain-containing protein 5A** | TMCO5A | 5.83 | 34,174 |
| **92** | TTHY_HUMAN | **Transthyretin** | TTR | 5.49 | 15,887 |
| **93** | Q9Y5T5_HUMAN | **Ubiquitin carboxyl-terminal hydrolase 16** | USP16 | 6.50 | 93,570 |
| **94** | Q9Y5T5-2_HUMAN | **Ubiquitin carboxyl-terminal hydrolase 16** (Isoform2) | USP16 | 6.50 | 93,499 |
| **95** | Q9Y5T5-3_HUMAN | **Ubiquitin carboxyl-terminal hydrolase 16** (Isoform3) | USP16 | 6.35 | 91,870 |
| **96** | Q9Y5T5-4_HUMAN | **Ubiquitin carboxyl-terminal hydrolase 16** (Isoform4) | USP16 | 6.36 | 58,397 |
| **97** | Q9Y5T5-5_HUMAN | **Ubiquitin carboxyl-terminal hydrolase 16** (Isoform5) | USP16 | 7.02 | 46,599 |
| **98** | Q6P097_HUMAN | **V-set domain-containing T-cell activation inhibitor 1** | VTCN1 | 5.20 | 30,878 |
| **99** | Q7Z7D3-2_HUMAN | **V-set domain-containing T-cell activation inhibitor 1** (isoform2) | VTCN1 | 5.07 | 18,275 |
| **100** | Q7Z7D3-3_HUMAN | **V-set domain-containing T-cell activation inhibitor 1** (isoform3) | VTCN1 | 6.95 | 9,875 |
| **101** | Q7Z7D3-4_HUMAN | **V-set domain-containing T-cell activation inhibitor 1** (isoforms4) | VTCN1 | 8.32 | 20,743 |
| **102** | ZN276_HUMAN | **Zinc finger protein 276** | ZNF276 | 8.87 | 67,219 |
| **103** | ZN775_HUMAN | **Zinc finger protein 775** | ZNF775 | 10.23 | 59,752 |
| **104** | ZN775_HUMAN | **Zinc finger protein 775** | ZNF775 | 10.23 | 59,752 |

**Figure 3S:** Electrophoresis profile of coelomic fluid after HTP purification procedure, **Protocol II**.

**Lanes:** **M:** Marker. **1:** coelomic fluid, positive control. **2:** HTP waste fractions; **3-7:** elution fractions at pH 8;

**8-9:**elution fractions at pH 10; **10-12:** elution fractions in water.

**Figure 4S:** Linear MALDI spectra of two elution fractions from **Protocol II.** (**A**) HTP elution fraction-**1** in H_2_O; (**B**) HTP elution fraction-**2** in H_2_O.

**Figure 5S:** Linear MALDI spectra of two fractions from **Protocol III_b_ PROT-BA.** (**A**) coelomic fraction before depletion procedure; (**B**) fraction after depletion procedure.

**Table 3S:** **PROT-BA (Protocol III_b_** ProteoPrep Blu Albumin and IgG depletion Medium, Sigma Aldrich**)**.

The identification of proteins was performed using the Protein Pilot Paragon Method. The MS/MS data were processed using a mass tolerance of 10 ppm and 0.2 Da for the precursor and fragment ions, respectively. **^a^**According to “UniProtKB” (http://www.uniprot.org/). **^b^**According to “Compute pI/MW” (http://web.expasy.org/compute_pi/).

|  | **Accession^a^** | **Protein Name^a^** | **Gene Name^a^** | **IP^b^** | **MW (Da)^a^** |
| --- | --- | --- | --- | --- | --- |
| **1** | E1B6W4_HUMAN | **Arginine/serine-rich coiled-coil protein 2** (fragment) | RSRC2 | 12.04 | 28,217 |
|  | H0YGP9_HUMAN | **Arginine/serine-rich coiled-coil protein 2** (fragment) | RSRC2 | 12.18 | 21,688 |
|  | RSRC2_HUMAN | **Arginine/serine-rich coiled-coil protein 2** (isoform1) | RSRC2 | 11.33 | 50,560 |
|  | RSRC2_HUMAN | **Arginine/serine-rich coiled-coil protein 2** (isoform2) | RSRC2 | 11.32 | 44,879 |
| **2** | KAP3_HUMAN | **cAMP-dependent protein kinase type II-beta regulatory subunit** | PRKAR2B | 4.82 | 46,302 |
| **3** | CARL2_HUMAN | **Capping protein, Arp2/3 and myosin-I linker protein 2** (isoform1) | CARMIL2 | 6.31 | 154,689 |
|  | CARL2_HUMAN | **Capping protein, Arp2/3 and myosin-I linker protein 2** (isoform1) | CARMIL2 | 6.11 | 148,208 |
| **4** | CAH14_HUMAN | **Carbonic anhydrase 14** | CA14 | 5.90 | 37,668 |
| **5** | CHD1_HUMAN | **Chromodomain-helicase-DNA-binding protein 1** (isoform1) | CHD1 | 6.68 | 196,688 |
|  | CHD1_HUMAN | **Chromodomain-helicase-DNA-binding protein 1** (isoform2) | CHD1 | 6.68 | 196,590 |
| **6** | CO1A1_HUMAN | **Collagen alpha-1(I) chain** | COL1A1 | 5.60 | 138,941 |
| **7** | CNDG2_HUMAN | **Condensin-2 complex subunit G2** (isoform1) | NCAPG2 | 6.43 | 130,960 |
|  | CNDG2_HUMAN | **Condensin-2 complex subunit G2** (isoform2) | NCAPG2 | 6.33 | 132,196 |
| **8** | CISH_HUMAN | **Cytokine-inducible SH2-containing protein** (isoform1) | CISH | 6.52 | 28,663 |
|  | CISH_HUMAN | **Cytokine-inducible SH2-containing protein** (isoform1B) | CISH | 7.01 | 29,288 |
|  | CISH_HUMAN | **Cytokine-inducible SH2-containing protein** (isoform1C) | CISH | 5.99 | 30,734 |
| **9** | DPYD_HUMAN | **Dihydropyrimidine dehydrogenase [NADP(+)]** (isoform1) | DPYD | 6.80 | 111,401 |
| **10** | SHLB1_HUMAN | **Endophilin-B1** (isoform1) | SH3GLB1 | 5.78 | 40,796 |
|  | SHLB1_HUMAN | **Endophilin-B1** (isoform2) | SH3GLB1 | 5.53 | 43,196 |
|  | SHLB1_HUMAN | **Endophilin-B1** (isoform3) | SH3GLB1 | 6.00 | 29,318 |
| **11** | CASR_HUMAN | **Extracellular calcium-sensing receptor** (isoform1) | CASR | 5.66 | 120,674 |
|  | CASR_HUMAN | **Extracellular calcium-sensing receptor** (isoform2) | CASR | 5.66 | 121,772 |
| **12** | FLIP1_HUMAN | **Filamin-A-interacting protein 1** (isoform1) | FILIP1 | 8.46 | 138,109 |
|  | FLIP1_HUMA | **Filamin-A-interacting protein 1** (isoform2) | FILIP1 | 8.03 | 134,859 |
|  | FLIP1_HUMAN | **Filamin-A-interacting protein 1** (isoform3) | FILIP1 | 8.15 | 109,300 |
| **13** | GLPK2_HUMAN | **Glycerol kinase 2** | GK2 | 5.57 | 60,594 |
| **14** | HCDH_HUMAN | **Hydroxyacyl-coenzyme A dehydrogenase, mitochondrial** (isoform1) | HADH | 8.88 | 34,294 |
|  | HCDH_HUMAN | **Hydroxyacyl-coenzyme A dehydrogenase, mitochondrial** (isoform2) | HADH | 9.34 | 42,140 |
| **15** | ILFT1_HUMAN | **Lamin tail domain-containing protein 1**(isoform1) | LMNTD1 | 9.18 | 43,408 |
|  | ILFT1_HUMAN | **Lamin tail domain-containing protein 1**(isoform2) | LMNTD1 | 9.15 | 33,043 |
|  | ILFT1_HUMAN | **Lamin tail domain-containing protein 1**(isoform3) | LMNTD1 | 9.21 | 36,755 |
|  | ILFT1_HUMAN | **Lamin tail domain-containing protein 1**(isoform4) | LMNTD1 | 9.17 | 41,395 |
|  | ILFT1_HUMAN | **Lamin tail domain-containing protein 1**(isoform5) | LMNTD1 | 9.06 | 45,891 |
| **16** | LRN4L_HUMAN | **LRRN4 C-terminal-like protein** | LRRN4CL | 6.07 | 25,262 |
| **17** | MBD1_HUMAN | **Methyl-CpG-binding domain protein 1** (isoform1) | MBD1 | 9.32 | 66,607 |
|  | MBD1_HUMAN | **Methyl-CpG-binding domain protein 1** (isoform2) | MBD1 | 9.39 | 64,677 |
|  | MBD1_HUMAN | **Methyl-CpG-binding domain protein 1** (isoform4) | MBD1 | 9.32 | 55,167 |
|  | MBD1_HUMAN | **Methyl-CpG-binding domain protein 1** (isoform5) | MBD1 | 9.49 | 61,265 |
|  | MBD1_HUMAN | **Methyl-CpG-binding domain protein 1** (isoform7) | MBD1 | 9.19 | 60,001 |
|  | MBD1_HUMAN | **Methyl-CpG-binding domain protein 1** (isoform8) | MBD1 | 9.58 | 59,158 |
|  | MBD1_HUMAN | **Methyl-CpG-binding domain protein 1** (isoform9) | MBD1 | 9.49 | 69,617 |
| **18** | MCCA_HUMAN | **Methylcrotonoyl-CoA carboxylase subunit alpha, mitochondrial** | MCCC1 | 7.66 | 80,473 |
|  | E9PHF7_HUMAN | **Methylcrotonoyl-CoA carboxylase subunit alpha, mitochondrial** | MCCC1 | 6.34 | 68,332 |
|  | E9PG35_HUMAN | **Methylcrotonoyl-CoA carboxylase subunit alpha, mitochondrial** (fragment) | MCCC1 | 6.86 | 66,468 |
| **19** | Q8IUX2_HUMAN | **Microtubule-associated protein 2** | MAP2 | 4.82 | 199,526 |
| **20** | MUC16_HUMAN | **Mucin-16** | MUC16 | 5.13 | 1,519,175 |
| **21** | MYOM2_HUMAN | **Myomesin-2** | MYOM2 | 5.82 | 164,896 |
| **22** | P210L_HUMAN | **Nuclear pore membrane glycoprotein 210-like** | NUP210L | 7.15 | 210,605 |
| **23** | ZDHC5_HUMAN | **Palmitoyltransferase ZDHHC5** (isoform1) | ZDHHC5 | 9.17 | 77,545 |
|  | ZDHC5_HUMAN | **Palmitoyltransferase ZDHHC5** (isoform2) | ZDHHC5 | 9.08 | 71,952 |
| **24** | PI51C_HUMAN | **Phosphatidylinositol 4-phosphate 5-kinase type-1 gamma** (isoform1) | PIP5K1C | 5.17 | 73,260 |
|  | PI51C_HUMAN | **Phosphatidylinositol 4-phosphate 5-kinase type-1 gamma** (isoform2) | PIP5K1C | 5.45 | 76,620 |
|  | PI51C_HUMAN | **Phosphatidylinositol 4-phosphate 5-kinase type-1 gamma** (isoform3) | PIP5K1C | 5.58 | 77,484 |
|  | PI51C_HUMAN | **Phosphatidylinositol 4-phosphate 5-kinase type-1 gamma** (isoform4) | PIP5K1C | 5.28 | 70,214 |
| **25** | ATP9B_HUMAN | **Probable phospholipid-transporting ATPase IIB** (isoform1) | ATP9B | 7.67 | 129,304 |
|  | ATP9B_HUMAN | **Probable phospholipid-transporting ATPase IIB** (isoform2) | ATP9B | 7.46 | 128,198 |
| **26** | DIAP2_HUMAN | **Protein diaphanous homolog 2** (isoform1) | DIAPH2 | 6.20 | 125,569 |
|  | DIAP2_HUMAN | **Protein diaphanous homolog 2** (isoform3) | DIAPH2 | 6.34 | 125,043 |
| **27** | FA65A_HUMAN | **Protein FAM65A** (isoform1) | FAM65A | 5.87 | 132,308 |
|  | FA65A_HUMAN | **Protein FAM65A** (isoform2) | FAM65A | 5.87 | 131,873 |
| **28** | PP14B_HUMAN | **Protein phosphatase 1 regulatory subunit 14B** | PPP1R14B | 4.75 | 15,911 |
|  | F5GXC4_HUMAN | **Protein phosphatase 1 regulatory subunit 14B** (fragment) | PPP1R14B | 8.93 | 5,082 |
|  | F5H2U0_HUMAN | **Protein phosphatase 1 regulatory subunit 14B** (fragment) | PPP1R14B | 4.40 | 8,103 |
| **29** | PCDGC_HUMAN | **Protocadherin gamma-A12** | PCDHGA12 | 4.90 | 100,955 |
| **30** | PPOX_HUMAN | **Protoporphyrinogen oxidase** | PPOX | 8.43 | 50,765 |
| **31** | PRXD1_HUMAN | **Putative prolyl-tRNA synthetase associated domain-containing protein 1** | PRORSD1P | 5.81 | 18,658 |
| **32** | ALBU_HUMAN | **Serum Albumin** | ALB | 5.92 | 69,367 |
| **33** | SPP2B_HUMAN | **Signal peptide peptidase-like 2B** | SPPL2B | 8.67 | 64,644 |
| **34** | PM14_HUMAN | **Splicing factor 3B subunit 6** | SF3B6 | 9.41 | 14,585 |
| **35** | SOCS3_HUMAN | **Suppressor of cytokine signaling 3** | SOCS3 | 8.97 | 24,770 |
| **36** | TRAK1_HUMAN | **Trafficking kinesin-binding protein 1** | TRAK1 | 5.59 | 106,040 |
|  | C9JC32_HUMAN | **Trafficking kinesin-binding protein 1** (fragment) | TRAK1 | 5.57 | 99,683 |
| **37** | GATA5_HUMAN | **Transcription factor GATA-5** | GATA5 | 9.17 | 41,299 |
| **38** | ZEP3_HUMAN | **Transcription factor HIVEP3** (isoform1) | HIVEP3 | 7.85 | 259,465 |
|  | ZEP3_HUMAN | **Transcription factor HIVEP3** (isoform2) | HIVEP3 | 7.85 | 259,336 |
| **39** | T200B_HUMAN | **Transmembrane protein 200B** | TMEM200B | 11.44 | 32,750 |
| **40** | UBP45_HUMAN | **Ubiquitin carboxyl-terminal hydrolase 45** (isoform1) | USP45 | 8.32 | 91,733 |
|  | UBP45_HUMAN | **Ubiquitin carboxyl-terminal hydrolase 45** (isoform3) | USP45 | 7.99 | 55,812 |
| **41** | F5H0J8_HUMAN | **Ubiquitin-associated protein 1** | UBAP1 | 5.02 | 55,084 |
| **42** | UCN3_HUMAN | **Urocortin-3** | UCN3 | 10.41 | 17,961 |
| **43** | B4DLE8_HUMAN | **Very large A-kinase anchor protein** | CRYBG3 | 5.10 | 330,633 |
| **44** | Z280A_HUMAN | **Zinc finger protein 280A** | ZNF280A | 9.03 | 60,816 |
| **45** | ZN442_HUMAN | **Zinc finger protein 442** (isoform1) | ZNF442 | 9.03 | 72,863 |
|  | B4DJ48_HUMAN | **Zinc finger protein 442** (isoform2) | ZNF442 | 9.23 | 64,877 |
| **46** | E7ER33_HUMAN | **Zinc finger protein 568** (isoform1) | ZNF568 | 8.58 | 74,369 |
| **47** | ZN638_HUMAN | **Zinc finger protein 638** (isoform1) | ZNF638 | 6.02 | 220,625 |
|  | ZN638_HUMAN | **Zinc finger protein 638** (isoform3) | ZNF638 | 6.05 | 218,246 |
| **48** | H3BS68_HUMAN | **Zinc finger protein 821** (fragment) | ZNF821 | 6.37 | 45,782 |
|  | H3BRD6_HUMAN | **Zinc finger protein 821** (fragment) | ZNF821 | 5.36 | 27,703 |
|  | ZN821_HUMAN | **Zinc finger protein 821** (isoform1) | ZNF821 | 6.14 | 46,794 |
|  | ZN821_HUMAN | **Zinc finger protein 821** (isoform2) | ZNF821 | 6.59 | 42,140 |

**Table 4S:** **MARS (Protocol III_a_** Multiple affinity removal spin cartridge - MARS”, Agilent Technologies, Milan, Italy, 5188-8825, specific for albumin and IgG depletion). The identification of proteins was performed using the Protein Pilot Paragon Method. The MS/MS data were processed using a mass tolerance of 10 ppm and 0.2 Da for the precursor and fragment ions, respectively. **^a^**According to “UniProtKB” (http://www.uniprot.org/). **^b^**According to “Compute pI/MW” (http://web.expasy.org/compute_pi/).

|  | **Accession^a^** | **Protein Name^a^** | **Gene Name^a^** | **IP^b^** | **MW (Da)^a^** |
| --- | --- | --- | --- | --- | --- |
| **1** | PHP14_HUMAN | **14 kDa phosphohistidine phosphatase** | PHPT1 | 5.65 | 13,833 |
| **2** | CH60_HUMAN | **60 kDa heat shock protein, mitochondrial** | HSPD1 | 5.70 | 61,055 |
| **3** | B8ZWD6_HUMAN | **Acyl-CoA-binding protein** | DBI | 6.12 | 10,044 |
| **4** | A16A1_HUMAN | **Aldehyde dehydrogenase family 16 member A1** | ALDH16A1 | 6.35 | 85,127 |
| **5** | FETUA_HUMAN | **Alpha-2-HS-glycoprotein/Fetuin** | AHSG | 5.43 | 39,325 |
| **6** | ANGT_HUMAN | **Angiotensinogen** | AGT | 5.87 | 53,154 |
| **7** | AN36C_HUMAN | **Ankyrin repeat domain-containing protein 36C** | ANKRD36C | 8.02 | 199,748 |
| **8** | ANT3_HUMAN | **Antithrombin-III** | SERPINC1 | 6.32 | 52,602 |
| **9** | ABCC9_HUMAN | **ATP-binding cassette sub-family C member 9** | ABCC9 | 7.05 | 174,223 |
| **10** | H3BUH8_HUMAN | **BAI1-associated protein 3** | BAIAP3 | 5.95 | 131,901 |
| **11** | PGBM_HUMAN | **Basement membrane-specific heparan sulfate proteoglycan core protein** | HSPG2 | 6.06 | 468,830 |
| **12** | BASI_HUMAN | **Basigin** | BSG | 5.39 | 42,200 |
| **13** | BCL9_HUMAN | **B-cell CLL/lymphoma 9 protein** | BCL9 | 8.99 | 149,290 |
| **14** | BCOR_HUMAN | **BCL-6 corepressor** | BCOR | 6.06 | 192,189 |
| **15** | BTBDI_HUMAN | **BTB/POZ domain-containing protein 18** | BTBD18 | 4.94 | 77,931 |
| **16** | CADH8_HUMAN | **Cadherin-8** | CDH8 | 4.55 | 88,253 |
| **17** | CAPS2_HUMAN | **Calcium-dependent secretion activator 2** | CADPS2 | 5.83 | 147,735 |
| **18** | CPNS2_HUMAN | **Calpain small subunit 2** | CAPNS2 | 5.51 | 27,660 |
| **19** | CATH_HUMAN | **Cathepsin H light chain** | CTSH | 8.35 | 37,394 |
| **20** | CERU_HUMAN | **Ceruloplasmin OS=Homo sapiens** | CP | 5.44 | 122,205 |
| **21** | C2D1B_HUMAN | **Coiled-coil and C2 domain-containing protein 1B** | CC2D1B | 5.18 | 94,224 |
| **22** | CC142_HUMAN | **Coiled-coil domain-containing protein 142** | CCDC142 | 6.58 | 81,643 |
| **23** | CO1A1_HUMAN | **Collagen alpha-1(I) chain** | COL1A1 | 5.60 | 138,941 |
| **24** | CO4A2_HUMAN | **Collagen alpha-2(IV) chain** | COL4A2 | 8.89 | 167,553 |
| **25** | CO3_HUMAN | **Complement C3** | C3 | 6.02 | 187,148 |
| **26** | KCRU_HUMAN | **Creatine kinase U-type, mitochondrial** | CKMT1A | 8.60 | 47,037 |
| **27** | DPP6_HUMAN | **Dipeptidyl aminopeptidase-like protein 6** | DPP6 | 5.94 | 97,588 |
| **28** | CD248_HUMAN | **Endosialin** | CD248 | 5.18 | 80,859 |
| **29** | ELF4_HUMAN | **ETS-related transcription factor Elf-4** | ELF4 | 5.41 | 70,730 |
| **30** | FIBB_HUMAN | **Fibrinogen beta chain** | FGB | 8.54 | 55,928 |
| **31** | FINC_HUMAN | **Fibronectin** | FN1 | 5.46 | 262,625 |
| **32** | FSIP2_HUMAN | **Fibrous sheath-interacting protein** | FSIP2 | 6.27 | 780,607 |
| **33** | GCP5_HUMAN | **Gamma-tubulin complex component 5** | TUBGCP5 | 5.58 | 118,321 |
| **34** | GEMI_HUMAN | **Geminin** | GMNN | 4.93 | 23,565 |
| **35** | H7BYP9_HUMAN | **Guanine nucleotide exchange factor DBS (Fragment)** | MCF2L | 6.98 | 89,107 |
| **36** | HXK2_HUMAN | **Hexokinase-2** | HK2 | 5.71 | 102,380 |
| **37** | SAP30_HUMAN | **Histone deacetylase complex subunit SAP30** | SAP30 | 9.28 | 23,306 |
| **38** | ASH1L_HUMAN | **Histone-lysine N-methyltransferase ASH1L** | ASH1L | 9.46 | 332,790 |
| **39** | KV312_HUMAN | **Ig kappa chain V-III region HAH** | IGKV3-20 | 4.85 | 12,557 |
| **40** | GP180_HUMAN | **Integral membrane protein GPR180** | GPR180 | 7.01 | 49,395 |
| **41** | ITAX_HUMAN | **Integrin alpha-X** | ITGAX | 6.19 | 127,829 |
| **42** | IL36B_HUMAN | **Interleukin-36 beta** (isoform 1) | IL36B | 9.60 | 18,522 |
|  | IL36B_HUMAN | **Interleukin-36 beta** (Isoform 2) | IL36B | 9.13 | 17,702 |
| **43** | K2C5_HUMAN | **Keratin, type II cytoskeletal 5** | KRT5 | 7.58 | 62,378 |
| **44** | KNG1_HUMAN | **Kininogen-1** | KNG1 | 6.34 | 71,957 |
| **45** | LMIP_HUMAN | **Lens fiber membrane intrinsic protein** (Isoform 2) | LIM2 | 9.64 | 19,674 |
| **46** | LRRK2_HUMAN | **Leucine-rich repeat serine/threonine-protein kinase 2** | LRRK2 | 6.35 | 286,103 |
| **47** | LIPA1_HUMAN | **Liprin-alpha-1** | PPFIA1 | 5.91 | 135,779 |
| **48** | HMHA1_HUMAN | **Minor histocompatibility protein HA-1** | ARHGAP45 | 5.76 | 124,614 |
| **49** | MEGF6_HUMAN | **Multiple epidermal growth factor-like domains protein 6** | MEGF6 | 5.94 | 161,185 |
| **50** | SYNE4_HUMAN | **Nesprin-4** | SYNE4 | 5.48 | 43,512 |
| **51** | NEMF_HUMAN | **Nuclear export mediator factor NEMF** | NEMF | 5.97 | 122,954 |
| **52** | NUFP1_HUMAN | **Nuclear fragile X mental retardation-interacting protein 1** | NUFIP1 | 9.21 | 56,300 |
| **53** | NACC2_HUMAN | **Nucleus accumbens-associated protein 2** | NACC2 | 5.64 | 62,837 |
| **54** | OXYR_HUMAN | **Oxytocin receptor** | OXTR | 9.63 | 42,772 |
| **55** | NR2E3_HUMAN | **Photoreceptor-specific nuclear receptor** | NR2E3 | 8.17 | 44,692 |
| **56** | PEAR1_HUMAN | **Platelet endothelial aggregation receptor 1** | PEAR1 | 6.40 | 110,666 |
| **57** | KCNH6_HUMAN | **Potassium voltage-gated channel subfamily H member 6** | KCNH6 | 6.55 | 109,925 |
| **58** | ATP9B_HUMAN | **Probable phospholipid-transporting ATPase IIB** | ATP9B | 7.67 | 129,304 |
| **59** | NRG3_HUMAN | **Pro-neuregulin-3, membrane-bound isoform** (Isoform 2) | NRG3 | 7.79 | 77,901 |
| **60** | PE2R1_HUMAN | **Prostaglandin E2 receptor EP1 subtype** | PTGER1 | 11.72 | 41,801 |
| **61** | PSA3_HUMAN | **Proteasome subunit alpha type-3** | PSMA3 | 5.19 | 28,433 |
|  | PSME2_HUMAN | **Proteasome activator complex subunit 2** | PSME2 | 5.54 | 27,402 |
| **62** | MCM10_HUMAN | **Protein MCM10 homolog** | MCM10 | 8.96 | 98,183 |
| **63** | WWC2_HUMAN | **Protein WWC2** | WWC2 | 5.40 | 133,891 |
| **64** | PCD16_HUMAN | **Protocadherin-16** | DCHS1 | 4.79 | 346,181 |
| **65** | YO011_HUMAN | **Putative transmembrane protein** | N/A | 8.96 | 13,671 |
| **66** | CU129_HUMAN | **Putative uncharacterized protein encoded by LINC00479 O** | LINC00479 | 8.28 | 15,208 |
| **67** | MEX3B_HUMAN | **RNA-binding protein MEX3B** | MEX3B | 6.44 | 58,832 |
| **68** | SASH1_HUMAN | **SAM and SH3 domain-containing protein 1** | SASH1 | 5.78 | 136,653 |
| **69** | WNK2_HUMAN | **Serine/threonine-protein kinase WNK2** | WNK2 | 5.79 | 242,676 |
| **70** | TRFE_HUMAN | **Serotransferrin** | TF | 6.81 | 77,064 |
| **71** | ALBU_HUMAN | **Serum albumin** | ALB | 5.92 | 69,367 |
| **72** | SH321_HUMAN | **SH3 domain-containing protein 21 O** | SH3D21 | 5.60 | 70,519 |
| **73** | SCNBA_HUMAN | **Sodium channel protein type 11 subunit alpha** (Isoform 3) | SCN11A | 8.44 | 200,415 |
| **74** | S28A3_HUMAN | **Solute carrier family 28 member 3** | SLC28A3 | 7.56 | 76,930 |
| **75** | STAT_HUMAN | **Statherin** | STATH | 8.01 | 7,304 |
| **76** | SVEP1_HUMAN | **Sushi, von Willebrand factor type A, EGF and pentraxin domain-containing protein 1** | SVEP1 | 5.32 | 390,170 |
| **77** | THYG_HUMAN | **Thyroglobulin** | TG | 5.40 | 304,790 |
| **78** | ZO2_HUMAN | **Tight junction protein ZO-2** | TJP2 | 6.96 | 133,958 |
| **79** | TOR4A_HUMAN | **Torsin-4A** | TOR4A | 9.98 | 46,914 |
| **80** | TCF15_HUMAN | **Transcription factor 15** | TCF15 | 9.97 | 20,816 |
| **81** | BACH2_HUMAN | **Transcription regulator protein BACH2** | BACH2 | 5.00 | 92,537 |
| **82** | TICRR_HUMAN | **Treslin** | TICRR | 9.00 | 210,857 |
| **83** | MOD5_HUMAN | **tRNA dimethylallyltransferase, mitochondrial** | TRIT1 | 8.35 | 52,725 |
| **84** | TUB_HUMAN | **Tubby protein homolog** (Isoform 1) | TUB | 8.88 | 55,651 |
|  | TUB_HUMAN | **Tubby protein homolog** (Isoform 2) | TUB | 9.56 | 62,091 |
| **85** | PTN14_HUMAN | **Tyrosine-protein phosphatase non-receptor type 14** | PTPN14 | 8.53 | 135,261 |
| **86** | UBAC2_HUMAN | **Ubiquitin-associated domain-containing protein 2** (Isoform 2) | UBAC2 | 9.21 | 38,964 |
| **87** | CX023_HUMAN | **Uncharacterized protein CXorf23** | CXorf23 | 9.71 | 83,871 |
| **88** | CSPG2_HUMAN | **Versican core protein** | VCAN | 4.43 | 372,820 |
| **89** | MIO_HUMAN | **WD repeat-containing protein mio** | MIOS | 6.29 | 98,584 |
| **90** | WIF1_HUMAN | **Wnt inhibitory factor 1** | WIF1 | 7.84 | 41,528 |
| **91** | YRDC_HUMAN | **YrdC domain-containing protein, mitochondrial** | YRDC | 8.90 | 29,328 |
| **92** | ZSC20_HUMAN | **Zinc finger and SCAN domain-containing protein 20** (Isoform 3) | ZSCAN20 | 6.01 | 117,470 |
| **93** | ZN775_HUMAN | **Zinc finger protein 775** | ZNF775 | 10.23 | 59,752 |
| **94** | ZZEF1_HUMAN | **Zinc finger ZZ-type and EF-hand domain-containing protein 1** | ZZEF1 | 5.62 | 331,075 |
| **95** | ZZZ3_HUMAN | **ZZ-type zinc finger-containing protein 3** | ZZZ3 | 5.48 | 102,023 |
